# Comparative Hessian Fly Larval Transcriptomics Provides Novel Insight into Host and Nonhost Resistance

**DOI:** 10.3390/ijms222111498

**Published:** 2021-10-25

**Authors:** Subhashree Subramanyam, Jill A. Nemacheck, Shaojun Xie, Ketaki Bhide, Jyothi Thimmapuram, Steven R. Scofield, Nagesh Sardesai

**Affiliations:** 1 Crop Production and Pest Control Research Unit, USDA-ARS, West Lafayette, IN 47907, USA; Jill.Nemacheck@usda.gov (J.A.N.); Steve.Scofield@usda.gov (S.R.S.); 2 Department of Entomology, Purdue University, West Lafayette, IN 47907, USA; 3 Bioinformatics Core, Purdue University, West Lafayette, IN 47907, USA; xie186@purdue.edu (S.X.); bhide@purdue.edu (K.B.); jyothit@purdue.edu (J.T.); 4 Department of Agronomy, Purdue University, West Lafayette, IN 47907, USA; 5 Corteva Agriscience, Johnston, IA 50131, USA; nagesh.sardesai@corteva.com

**Keywords:** *Mayetiola destructor*, *Triticum aestivum*, *Brachypodium distachyon*, Diptera, plant–insect interaction, RNA-Seq, gall midge, salivary effectors, compatible interaction, incompatible interaction

## Abstract

The Hessian fly is a destructive pest of wheat. Employing additional molecular strategies can complement wheat’s native insect resistance. However, this requires functional characterization of Hessian-fly-responsive genes, which is challenging because of wheat genome complexity. The diploid *Brachypodium distachyon* (Bd) exhibits nonhost resistance to Hessian fly and displays phenotypic/molecular responses intermediate between resistant and susceptible host wheat, offering a surrogate genome for gene characterization. Here, we compared the transcriptomes of Biotype L larvae residing on resistant/susceptible wheat, and nonhost Bd plants. Larvae from susceptible wheat and nonhost Bd plants revealed similar molecular responses that were distinct from avirulent larval responses on resistant wheat. Secreted salivary gland proteins were strongly up-regulated in all larvae. Genes from various biological pathways and molecular processes were up-regulated in larvae from both susceptible wheat and nonhost Bd plants. However, Bd larval expression levels were intermediate between larvae from susceptible and resistant wheat. Most genes were down-regulated or unchanged in avirulent larvae, correlating with their inability to establish feeding sites and dying within 4–5 days after egg-hatch. Decreased gene expression in Bd larvae, compared to ones on susceptible wheat, potentially led to developmentally delayed 2nd-instars, followed by eventually succumbing to nonhost resistance defense mechanisms.

## 1. Introduction

The Hessian fly, *Mayetiola destructor* (Say), belonging to the gall midge family Cecidomyiidae (order: Diptera) is an obligate pest of host bread wheat (*Triticum aestivum*) causing severe economic losses [1,2]. The newly hatched 1st-instar (neonate) larvae, which are assumed to be nonfeeding, migrate from the leaves to the plant crown, feed voraciously, undergo various developmental stages (1st, 2nd, 3rd-instars and pupae), and complete their life cycle in as few as 28–30 days. The recognition of insect avirulence (*avr*) gene product by plant Hessian fly resistance (*H*) gene product [3] yields an incompatible interaction (avirulent larvae, resistant plants). In this interaction, the wheat seedling mounts *H*-gene-mediated resistance, resulting in increased accumulation of defense proteins [4,5,6,7] that disrupt larval midgut microvilli [8]. The avirulent larvae die within 4–5 days after egg-hatch (DAH), and the resistant plants show normal growth. Lack of corresponding *H* gene in plants yields a compatible interaction (virulent larvae, susceptible plants). In this interaction, within three days of larval attack, the virulent larvae establish permanent feeding sites, alter host plant physiology and metabolic pathways [9], and upregulate susceptibility-associated genes [10,11,12], resulting in the formation of a nutritive tissue [13] that provides a nutrient-rich diet [12,14,15]. The larvae extra-orally produce a plant-cell-wall-degrading enzyme that degrades wall inulin polymers, making the plant a nutrient sink [16]. The virulent larvae complete their development, and the susceptible plant has a stunted phenotype, leading to the eventual death of the wheat seedling [17].

Planting resistant wheat cultivars is the most effective and economic approach to reduce the damage caused by Hessian fly [18,19]. A total of 37 *H* genes (*H1*-*H36* and *Hdic*) have been identified from wheat and its wild relatives [20,21,22]. To date, a total of 16 Hessian fly biotypes (A to O and Great Plains (GP)) have been documented based on their responses to wheat differentials harboring different *H* genes. Extensive deployment of resistant wheat lines exerts enormous selection pressure on Hessian fly, resulting in the development of virulent biotypes that can overcome plant resistance [23,24]. It is therefore imperative to develop alternate molecular strategies, such as developing wheat lines that overexpress or negatively regulate candidate Hessian-fly-responsive genes to enhance and complement native *H* gene resistance. However, such an approach requires prior functional characterization of the candidate Hessian-fly-responsive genes, which in hexaploid wheat has been very challenging owing to its huge genome size and complexity. 

To overcome this barrier, we recently explored and documented the suitability of utilizing less complex genomes for undertaking functional characterization of candidate Hessian-fly-responsive genes [25,26,27]. The small grass species *Brachypodium distachyon* (Bd), or the purple false brome, is one such model genome with a small size (diploid, 355 Mb), short life cycle, and availability of genetic resources [28,29], besides serving as a model system for nonhost resistance for several pathogens [30] and insects [31]. Recently, we investigated the physical and molecular responses of Bd plants to Hessian fly larval infestation [25,32] and proposed its suitability as a surrogate genome for functional studies [25]. Bd plants challenged with Hessian fly larvae exhibit type I and II nonhost resistance with physical and metabolic characteristics intermediate to both resistant and susceptible host wheat with no yield penalty [32]. Most larvae on Bd plants die within a few days of feeding, resembling the larval phenotypic responses observed in resistant wheat. However, 25% of the larvae grow to the small 2nd-instar stage, surviving up to 41 days longer than the avirulent larvae feeding on resistant wheat, but unable to complete their development [32]. Global transcriptome analysis of Hessian-fly-infested Bd plants at 1, 3, and 5 DAH revealed the presence of competing defense strategies with differential expression of genes and associated biological pathways resembling both the resistant and susceptible host wheat [25]. Since Bd plants exhibited intermediate physical and metabolic responses between resistant and susceptible host wheat, we hypothesized that the transcriptional changes in the larvae feeding on nonhost Bd plants will also be intermediate between avirulent and virulent Hessian fly larvae feeding on resistant and susceptible wheat, respectively. 

In this study, we employed the Next Generation Sequencing approach to compare and contrast global changes in gene expression in Hessian fly larvae. Illumina RNA-Sequencing (RNA-Seq) was performed on Biotype L larvae feeding on nonhost Bd plants and on avirulent and virulent Biotype L larvae feeding on resistant and susceptible host wheat, respectively. Although a previous study has been undertaken characterizing the differentially expressed genes (DEGs) in Hessian fly larvae, the study was carried out using the Great Plains biotype and only compared transcriptomes of larvae feeding on resistant and susceptible wheat plants, but not nonhost plants [33]. Our objective was to use the comparative transcriptomics data to understand the molecular mechanisms that are employed by the Hessian fly larvae when they are challenged with different biotic factors presented by resistant and susceptible host wheat and nonhost Bd plants. Our study is expected to enrich our knowledge base by providing an insight into molecular adaptive strategies employed by Hessian fly larvae to counter nonhost resistance. 

## 2. Results and Discussion

RNA-Seq identified several transcriptional changes occurring in Biotype L larvae feeding on both host wheat and nonhost Bd plants. It was necessary to investigate comparisons between the Biotype L larvae on wheat since the previous study [33] on virulent and avirulent larvae feeding on susceptible (Newton) and resistant (Molly) host wheat, respectively, used the GP (Great Plains) biotype, making a direct comparison with transcriptome changes in Biotype L larvae on nonhost Bd less ideal. Therefore, our current study attempts to directly compare the transcriptome of Biotype L larvae feeding on host wheat and nonhost Bd plants. 

### 2.1. Overview of Transcriptome of Larvae Feeding on Host Wheat and Nonhost Bd

**Figure 1:**
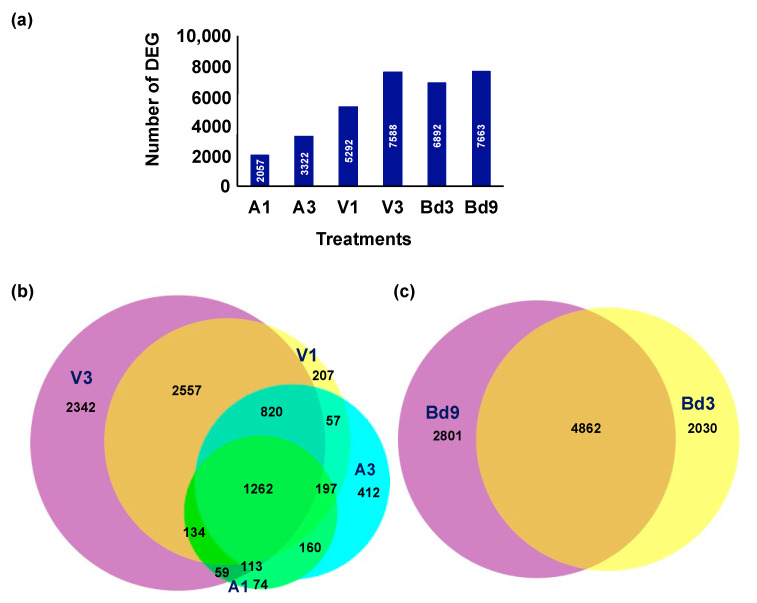
Differential expression of genes in Hessian fly larvae feeding on host wheat and nonhost *Brachypodium distachyon* (Bd) plants. Differentially expressed genes (DEG) were identified from avirulent (A) and virulent (V) Biotype L Hessian fly larvae feeding on host wheat at 1 (A1, V1) and 3 (A3, V3) days after egg hatch (DAH) as compared to the neonates, and Biotype L larvae feeding on nonhost Bd plants at 3 (Bd3) and 9 (Bd9) DAH as compared to neonates. (**a**) Number of statistically significant DEG (with log2 expression >1 or <−1 and adjusted *p*-value < 0.05) in A1, A3, V1, V3, Bd3, and Bd9 larvae. (**b**) Venn diagram depicting shared and uniquely expressed DEG between avirulent and virulent Biotype L larvae feeding on host wheat at 1 and 3 DAH. (**c**) Venn diagram depicting shared and uniquely expressed DEG between Biotype L larvae feeding on nonhost Bd plants at 3 and 9 DAH.

To reveal global transcriptome changes, RNA-Seq analysis was carried out with RNA extracted from avirulent (A1 and A3) and virulent (V1 and V3) larval samples collected from host wheat at 1 and 3 DAH and from larvae feeding on nonhost Bd plants at 3 (Bd3) and 9 (Bd9) DAH. We chose to compare the larvae feeding on Bd plants at 3 and 9 DAH instead of 1 DAH (Bd1) because our previous transcriptome study on the nonhost Bd plants showed that the response of Bd plants to Hessian fly larvae at 1 DAH for defense proteins, transcription factors, signaling kinases, and secondary metabolites resembled the resistant host wheat at 1 DAH, with more differences being observed in later time points of 3 and 5 DAH in Bd plants [25]. Sequencing was also carried out with the RNA isolated from the neonate larval samples collected from wheat (N0) and Bd (BdN) plants and used as controls. The high-throughput sequencing data have been submitted to NCBI Sequence Read Archive database public repository (PRJNA766660). Illumina sequencing yielded a total of 1,259,643,006 and 2,148,370,023 clean reads, with an average of 70 to 98 and 194 to 271 million reads for each transcriptome for larvae feeding on host and nonhost plants, respectively (Table S1). Mapping rates of the quality trimmed and filtered reads to the *M. destructor* genome reference ranged from 55 to 75 and 162 to 232 million reads per treatment, with 79% and 61% uniquely mapped for larvae feeding on host and nonhost, respectively (Table S1). Principal component analysis of read counts revealed a good correlation within larval samples feeding on host wheat (Figure S1a) and Bd nonhost plants (Figure S1b), demonstrating high consistency among the biological replicates. A total of 20,148 genes were identified from *M. destructor* that were functionally annotated against the nr database and three insect genomes. A total of 14,079 (69.88%), 12,250 (60.80%), 11,734 (58.24%), and 11,625 (57.70%) genes showed blast hits to nr database, *Aedes aegypti*, *Anopheles gambiae*, and *Drosophila melanogaster* genomes, respectively. Since the blast hits with the nr database did not yield annotations of gene descriptions for several of the differentially expressed genes (DEGs), the three additional reference insect genomes were selected. These three insect species were selected for annotation because they are also Dipterans and their genomes are well-annotated. Hessian fly larval attack led to significant differential expression (>2-fold-change, adjusted *p* value < 0.05) of genes in larvae feeding on host wheat and nonhost Bd plants. A total of 2057, 3322, 5292, 7588, 6892, and 7663 differentially expressed genes (DEGs) were identified in A1, A3, V1, V3, Bd3, and Bd9 samples as compared to neonates (N0 on host and BdN on nonhost), respectively (Figure 1a). A much higher number of DEGs were observed in virulent Biotype L larvae feeding on host plants both at 1 (V1) and 3 (V3) DAH time points as compared to those of avirulent larvae at the same time points (Figure 1a). Within virulent larvae, V3 larval transcriptome revealed a higher number of differentially expressed transcripts as compared to V1 larvae (Figure 1b). This is expected, as the virulent larvae are actively feeding and growing in contrast to the avirulent larvae, which are unable to feed and do not survive beyond 4–5 DAH. The number of DEGs from larval samples collected from nonhost Bd plants at 3 and 9 DAH were comparable with those seen in V1 and V3 samples, indicating similarities in transcriptional responses between these larvae. The number of DEGs was also higher in the Bd9 larvae as compared to Bd3 (Figure 1a), possibly as some larvae continue to feed and grow. Although the majority of larvae feeding on nonhost Bd plants die within a few days of egg hatch, a few larvae develop into developmentally delayed 2nd-instar larvae (smaller white), some of which prolong their survival up to 45 DAH [32]. A Venn diagram revealed a higher number of common transcripts within virulent and avirulent larvae than between them (Figure 1b), suggesting differences in transcriptional response between both larval types. Bd3 and Bd9 larvae also revealed a higher number of common transcripts than unique ones (Figure 1c). 

### 2.2. Validation of RNA-Seq Expression

To validate the RNA-Seq expression results, a total of 10 up- or down-regulated genes were selected to carry out qRT-PCR using larvae feeding on host wheat and nonhost Bd over a course of time. The expression data were highly correlated (Table S2). The Pearson correlation coefficient for the expression results between the two methods was 0.91 (Figure S2a) and 0.94 (Figure S2b) for host wheat and nonhost Bd plants, respectively, confirming the validity of the RNA-Seq data. 

### 2.3. Larval Phenotypes on Host Wheat and Nonhost Bd

**Figure 2:**
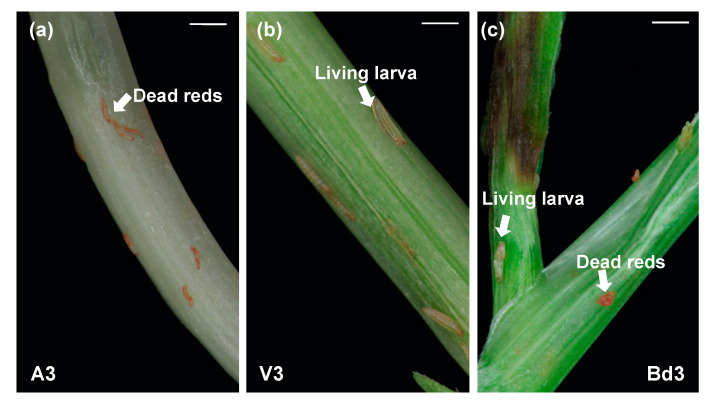
Phenotypic response of host wheat and nonhost Bd plants to Hessian fly larval feeding. (**a**) Biotype L-infested Iris wheat line showing host resistance resulting in avirulent larvae (A3) dying within 3 DAH and appearing as dead reds. (**b**) Biotype L-infested susceptible Newton wheat line with virulent live larvae (V3) in 2nd-instar stage. (**c**) Biotype L-infested Bd harboring both living larvae (2nd-instars) and dead reds (Bd3) by 3 DAH. Scale bars: 500 μm.

In susceptible wheat, within 3 days of larval attack, salivary secretions from the virulent larvae alter host plant metabolism [9], degrade cell walls [16], induce cell wall permeability [34], and convert the entire crown tissue into a nutrient sink [13], making the plant environment conducive for larvae to feed and develop. On the contrary, in response to larval attack, the resistant wheat plant mounts adequate defenses that prevent the avirulent larvae from feeding and altering host plant physiology. Phenotypically, these physiological changes within the host wheat impact the growth and development of larvae. By 3 DAH, the avirulent larvae (A3) appear shriveled as dead reds and do not develop beyond the 1st-instar stage (Figure 2a), while the virulent larvae (V3) appear as white, actively developing 2nd-instars (Figure 2b). Bd plants harbor both dead reds and small white 2nd-instar larvae by 3 DAH (Figure 2c). These small white larvae are able to survive the nonhost plant defense responses for up to 45 DAH [25,32]. Since by 3 DAH, phenotypic and molecular changes are distinct in larvae feeding on both host wheat and nonhost Bd plants, we further investigated the comparative transcriptional changes occurring in A3, V3, and Bd3 larvae to gain insight into differences and similarities between larval responses when feeding on host vs. nonhost.

### 2.4. Identification and Clustering of Differentially Expressed Genes (DEGs)

**Figure 3:**
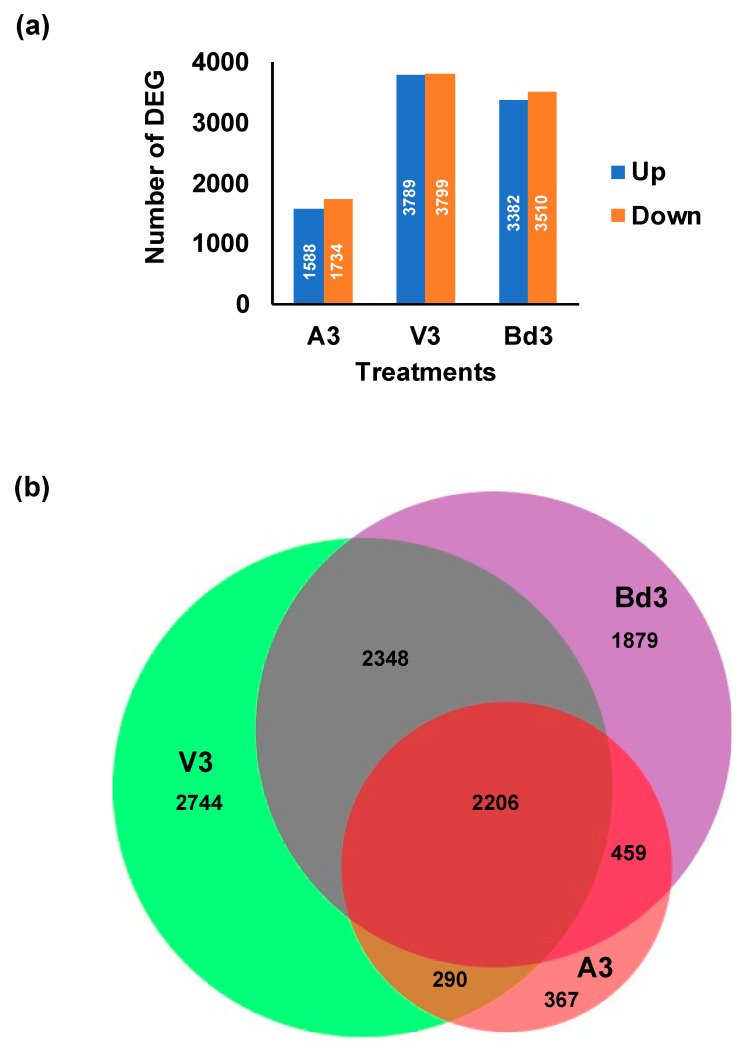
Up- and down-regulated differentially expressed genes (DEG) in larvae at 3 days after hatch (DAH). (**a**) The number of DEG (with log2 expression >1 or <−1 and adjusted *p*-value < 0.05) that showed up-regulation (blue bars) and down-regulation (orange bars) in avirulent (A3) and virulent (V3) larvae feeding on host wheat at 3 DAH and in larvae (Bd3) feeding on nonhost Bd plants at 3 DAH. (**b**) Venn diagram showing DEG that are uniquely expressed and common between A3, V3, and Bd3 larvae.

An equal number of up- and down-regulated genes were observed within each of the three treatments (Figure 3a). However, V3 and Bd3 larvae showed almost double the number of total up- or down-regulated DEGs as compared to A3 (Figure 3a). While all three samples had DEGs unique to the treatments, they shared a higher number (2206) of DEGs common between all three treatments (Figure 3b). V3 and Bd3 shared a much higher number of common DEGs (2348) between them as compared with Bd3 and A3 (Figure 3b). A3 larvae shared a higher number of common DEGs (459) with Bd3 as compared with V3 (290 DEGs) larvae (Figure 3b). 

**Figure 4:**
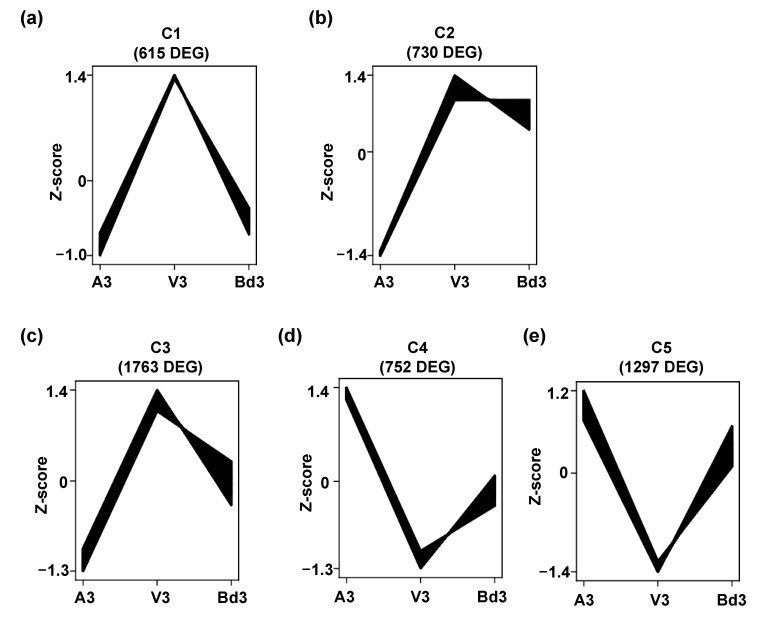
Cluster profiles of differentially expressed genes (DEG). Based on the transcript abundance and expression trends, the DEG identified from avirulent (A3), virulent (V3), and Bd3 larvae 3 days after hatch (DAH) were clustered into five groups. (**a**) C1 cluster with 615 DEG; (**b**) C2 cluster with 730 DEG; (**c**) C3 cluster with 1763 DEG; (**d**) C4 cluster with 752 DEG; (**e**) C5 cluster with 1297 DEG.

Based on the transcript accumulation levels in A3, V3, and Bd3 samples, the DEGs were grouped into five clusters (Figure 4, Table S3). In all five clusters, A3 and V3 showed contrasting expression profiles, with the DEGs in C1 (615 genes), C2 (730 genes), and C3 (1763) genes having negative Z-scores in A3 but positive Z-scores in V3 (Figure 4a–c). This trend was reversed in clusters C4 (752 genes) and C5 (1297 genes), where A3 had positive Z-scores and V3 had negative Z-scores (Figure 4d,e). In C1 (Figure 4a) and C2 (Figure 4b) clusters, Z-score values of Bd3 DEGs were close to A3 and V3 Z-score values, respectively. The cluster profiles C3 (Figure 4c), C4 (Figure 4d), and C5 (Figure 4e) clearly indicate that the levels of transcripts in Bd3 were intermediate between A3 and V3 transcriptome, mirroring the expression trends documented in Bd nonhost plants showing molecular responses intermediate between resistant and susceptible host wheat [25,32].

### 2.5. Differential Regulation of Specific Functional Categories

**Figure 5:**
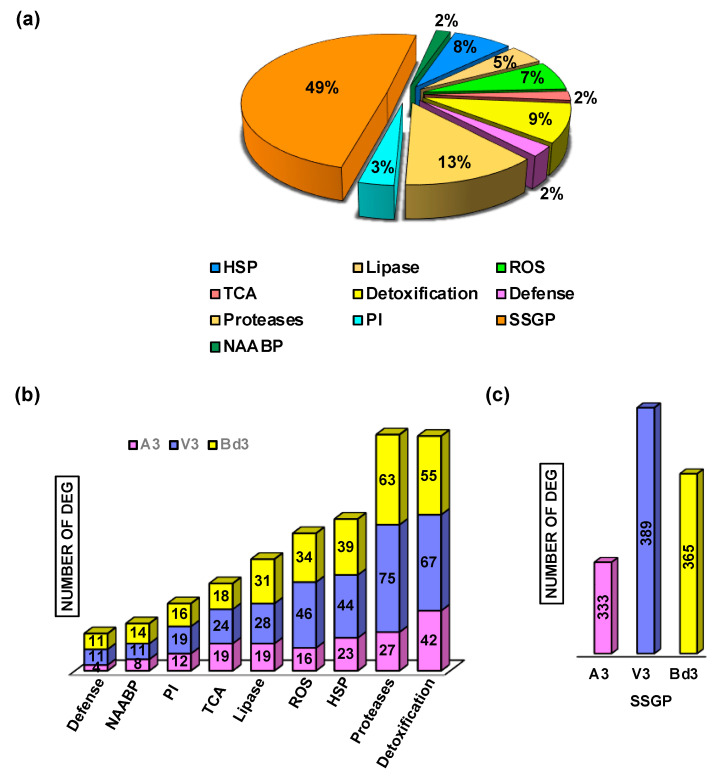
Differentially expressed genes (DEG) representing diverse functional categories. (**a**) Pie chart representing the fractions of functional categories representing the transcriptome of the avirulent (A3), virulent (V3), and Bd3 larvae. The categories are: Heat Shock Proteins (HSP), Lipase, Reactive Oxygen Species (ROS), Tricarboxylic Acid (TCA) pathway, Detoxification, Defense, Proteases, Protease Inhibitors (PI), Secreted Salivary Gland Proteins (SSGP), and Nonessential Amino Acid Biosynthetic Pathway (NAABP). (**b**) The number of DEG in A3 (pink), V3 (purple), and Bd3 (yellow) larval transcriptomes for genes representing different functional categories. (**c**) The number of DEG in A3 (pink), V3 (purple), and Bd3 (yellow) larval transcriptomes encoding SSGP.

To identify and compare the functional categories of genes that are differentially regulated in larvae feeding on host vs. nonhost, GO and KEGG enrichment analysis was performed with DEGs identified from A3 (Table S4), V3 (Table S5), and Bd3 (Table S6) larvae as compared to neonates from host wheat and nonhost Bd plants. The enrichment analysis revealed differential regulation of various biological pathways belonging to diverse biological processes (Figure S3), molecular function (Figure S4), cellular component (Figure S5), and KEGG pathways (Figure S6). Based on the enrichment analysis, we further investigated the transcriptional changes associated with the functional categories that are well-documented to play significant roles in the development of elegant defense and stress-mitigation systems in insects to sequester plant chemical toxins and suppress/counter plant defense responses. These categories include the Secreted Salivary Gland Proteins (SSGP) (49%), which constituted the largest fraction of transcripts, followed by genes encoding proteases (13%), detoxification enzymes (9%), heat shock proteins (8%), and enzymes leading to oxidative burst producing Reactive Oxygen Species (ROS) (7%) (Figure 5a). A small percentage of genes encoding lipases (5%), protease inhibitors (3%), and defense proteins, citric acid cycle enzymes, and nonessential amino acids biosynthetic pathway (NAABP) (2%) were also observed (Figure 5a). Within most of these functional categories, A3 larvae revealed the lowest number of transcripts (Figure 5b,c). V3 larvae exhibited the highest number of genes for most functional categories as compared to Bd3 larvae, which showed an intermediate number between A3 and V3 (Figure 5b,c), except for genes encoding lipases and NAABP, where Bd3 larvae showed a slightly higher number of DEGs than V3 larvae (Figure 5b), and for genes encoding defense proteins, with equal numbers in both V3 and Bd3 larvae (Figure 5b). Each of these functional categories were further dissected to reveal the family/class of genes being up- or down-regulated. 

#### 2.5.1. Regulation of Detoxification Enzymes

**Figure 6:**
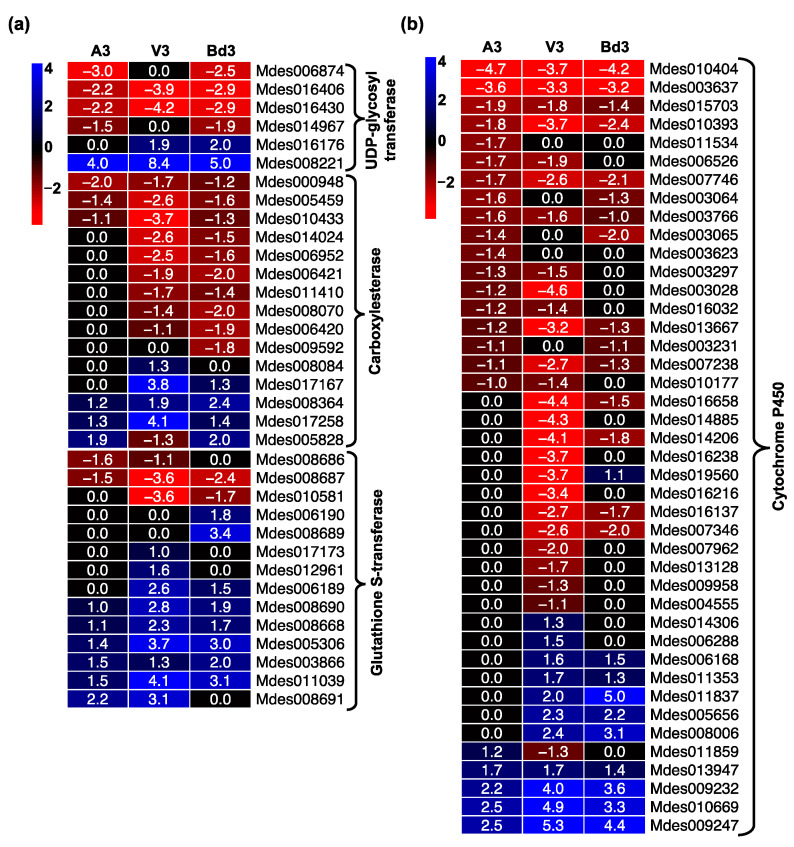
Regulation of detoxification enzymes in Hessian fly larvae 3 days after hatch (DAH). Heatmaps depict expression profiles of genes encoding detoxification enzymes in avirulent (A3), virulent (V3), and Bd3 larval transcriptomes. (**a**) Differential expression of genes encoding UDP-glycosyltransferase, carboxylesterase, and glutathione S-transferase enzymes. (**b**) Differential expression of genes encoding cytochrome P450 proteins. Log2 fold-change RNA-Seq values are shown within each cell of the heatmap. Blue represents up-regulated genes, and red represents the down-regulated genes, while genes not differentially expressed at a particular time-point are indicated in black.

Plants produce numerous defense toxins/allelochemicals that have detrimental effects on insect growth, reproduction, and survival [35,36,37]. Phytophagous insects have developed a complex detoxification system, comprised of a group of enzymes that metabolize these toxins and prevent damage of biological molecules within the gut. These include genes encoding carboxylestarase, cytochrome P450 (CYP450), glutathione S-transferase (GST), and uridine diphosphate (UDP)-glycosyltransferase (UGT) [38,39]. Increased accumulation of transcripts encoding these detoxification enzymes have been documented previously in other insects [40], as well as virulent Hessian fly larvae feeding on susceptible host wheat [33,41,42,43]. The transcriptome of A3, V3, and Bd3 larvae revealed differential expression of all four detoxification enzyme classes with 6, 15, 14, and 42 DEGs encoding UGT, carboxylesterase, GST, and CYP450, respectively (Figure 6). In all three samples, the expression trends for DEGs encoding these enzymes were very similar (Figure 6a,b). Many of the DEGs were not significantly expressed, and only a few were up- or down-regulated in A3 larvae (Figure 6a,b). Overall, in Bd3 and V3, approximately half the genes were down-regulated or up-regulated. Barring a few, most genes exhibited lowest, highest, and intermediate transcript abundance in A3, V3, and Bd3, respectively (Table S7). A larger repertoire of genes belonging to the cytochrome P450 class was observed in the V3 larval samples feeding on host wheat vs. nonhost Bd plants as compared to the other enzymes (Figure 6b). Most of the DEGs encoding GST (Figure 6a) were significantly up-regulated in A3, V3, and Bd3 larvae as compared to the other enzymes, while most DEGs encoding carboxylesterases and cytochrome P450s (Figure 6a,b) were down-regulated. Our RNA-Seq results for DEGs belonging to the cytochrome P450 class revealed that the avirulent larvae had higher transcript abundance of these genes than V3 and Bd3. A previous transcriptome study for the GP Hessian fly biotype revealed 8–10% more genes encoding cytochrome P450s had greater transcript abundance in avirulent larvae feeding on resistant plants as compared to virulent larvae feeding on susceptible plants [33]. However, amongst up-regulated genes, around 26% of cytochrome P450 genes were up-regulated in V3 compared to 12% in A3 and 24% in Bd3, suggesting that virulent larvae and larvae on Bd plants are able to detoxify more plant-derived allelochemicals than the avirulent larvae can. This was also true in the case of up-regulated genes of the GST, carboxylesterase, and UGT classes of DEGs, providing strong evidence that these genes likely contribute to the adaptation strategies for countering stress imposed due to ingestion of plant defense toxic molecules, eventually allowing Bd larvae to prolong their survival and V3 larvae to complete their development.

#### 2.5.2. Gene Expression Profiles of Proteases and Protease Inhibitors

**Figure 7:**
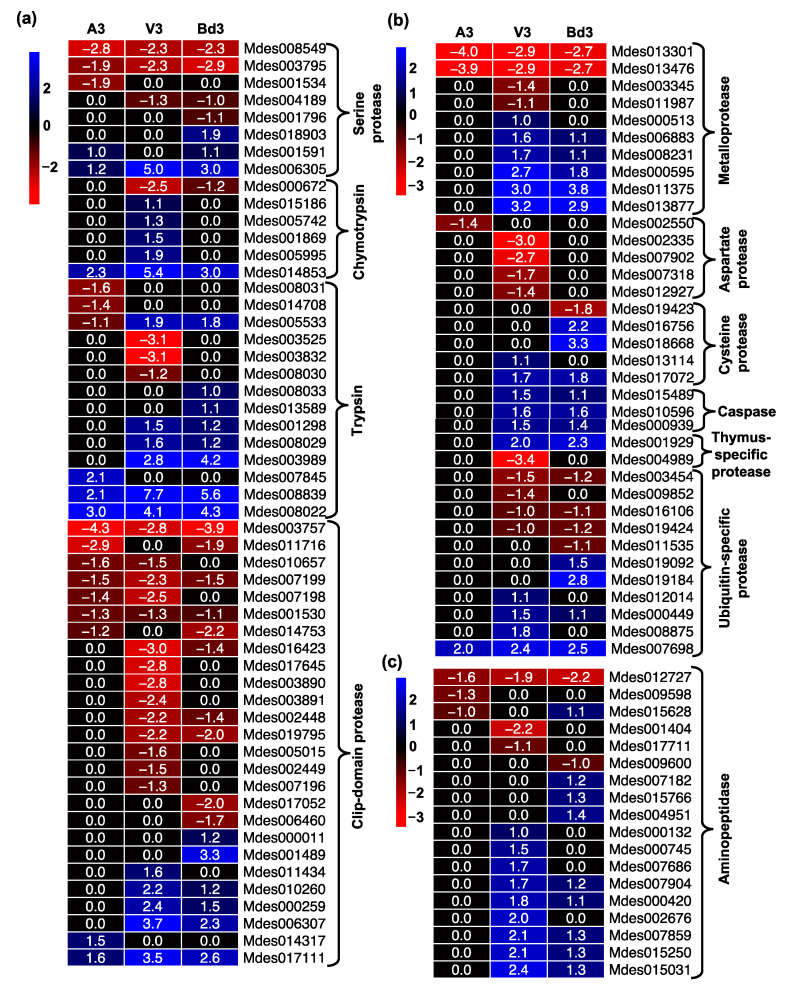
Differential expression of proteases in Hessian fly larvae 3 days after hatch (DAH). Heatmaps depict expression profiles of genes encoding several families of proteolytic enzymes in avirulent (A3), virulent (V3), and Bd3 larval transcriptomes. (**a**) Differential expression of genes encoding serine proteases including chymotrypsins, trypsins, and clip-domain proteases. (**b**) Expression of genes encoding metalloproteases, aspartate protease, cysteine protease, caspase, thymus-specific proteases, and ubiquitin-specific proteases. (**c**) Differential expression of genes encoding aminopeptidases. Log2 fold-change RNA-Seq values are shown within each cell of the heatmap. Blue represents up-regulated genes, and red represents the down-regulated genes, while genes not differentially expressed at a particular time-point are indicated in black.

Phytophagous insects produce a diverse array of proteases that play a crucial role in several physiological and biochemical processes, digestion of ingested proteins, and absorption of nutrients [44]. These proteases are classified into four main groups: (i) serine proteases, (ii) metalloproteases, (iii) aspartate proteases, and (iv) cysteine proteases [45]. A3, V3, and Bd3 larvae showed significant expression of genes belonging to all four groups of proteases (Figure 7). The serine proteases constitute a large family of proteolytic enzymes in insects. While chymotrypsin and trypsin are the major digestive serine proteases [46], the clip-domain serine proteases play an important role in insect development and immunity [47,48]. Hessian fly larvae feeding on host and nonhost plants showed differential regulation of 54 serine proteases including chymotrypsin, trypsin, and clip-domain classes (Figure 7a). Most of the chymotrypsins were up-regulated in V3 larvae, including *MDP1E* (Mdes014853) identified from GP biotype [49]. The *MDP1A* to *F* gene group has been shown to be significantly expressed in all Hessian fly larval stages of development with the highest transcripts accumulating in 4-day-old larvae [50]. The Mdes014853 gene (*MDP1E*) was also significantly up-regulated in A3 and Bd3 larvae (Figure 7a, Table S7). However, while it was expressed as high as 42-fold in V3 larvae, A3 and Bd3 larvae showed only 5–8-fold up-regulation (Table S7). Unlike chymotrypsin, a similar number of genes encoding trypsin protein were up-regulated in Bd3 compared to V3, but these were more than twice the number in A3 larvae (Figure 7a, Table S7). The transcript abundance for most of the trypsin genes was lower in Bd3 and A3 larvae as compared to V3, except for Mdes003989, which exhibited transcript levels several folds higher in Bd3 (18.6-fold) as compared to V3 (6.8-fold) larvae. This suggests that unlike Bd3, the digestive processes in V3 involves a higher repertoire of serine proteases that includes both chymotrypsin and trypsin. Chymotrypsin and trypsin serine proteases are demonstrated to be the major digestive enzymes in the gut of Hessian fly larvae [49]. Fewer up-regulated genes and lower expression levels in A3 larvae likely result in poor protease activity, which is correlated with the unsuccessful attempt to establish feeding sites by the avirulent larvae at 3 DAH [6]. In addition to trypsin and chymotrypsin, several genes encoding clip-domain proteases were also significantly up-regulated in V3 and Bd3 larvae that were not observed in A3 larvae (Figure 7a). Clip-domain proteases are involved in mediating protein–protein interactions or regulating a serine protease cascade that activates synthesis of antimicrobial peptides and prophenoloxidase [51,52], thus suggesting that these proteins are possibly involved in innate immunity in V3 and Bd3 larvae. In addition, clip-domain proteases are crucial in larval-pupal molting [48]. V3 larvae have several genes up-regulated that probably play a role in the growth and development of the larvae into pupae, while the Bd3 larvae had lower levels of expression for these genes that may account for why the larvae on Bd plants have a prolonged 2nd-instar phase without ever reaching pupation. A significant number of genes encoding other nondigestive proteases, including 10 metalloproteases, 5 aspartate proteases, and 8 cysteine proteases, were also differentially expressed (Figure 7b, Table S7). Bd3 and V3 larvae exhibited a comparable number of differentially expressed metalloproteases; however, the transcript levels of these genes were mostly lower in Bd3 as compared to V3 larvae (Figure 7b). Interestingly, while genes encoding aspartate protease were down-regulated in V3, none of these were differentially expressed in either Bd3 or A3 larvae (Figure 7b). Cysteine proteases were mostly up-regulated in Bd3, whereas they did not differ in expression in A3, while expression of genes encoding cysteine proteases, including caspase, a conserved family of cysteine proteases [53], was similar in V3 and Bd3 larvae but not differentially expressed in A3 larvae (Figure 7b). Bd3 and V3 larvae also showed similar profiles for genes encoding thymus-specific proteases and ubiquitin-specific proteases, with a comparable number of DEGs and expression levels. Genes encoding aminopeptidases, another class of proteases [54], also showed similar profiles in V3 and Bd3 larvae, with most not being differentially expressed in A3 larvae (Figure 7c). Levels of expression of these genes in Bd3 were lower than in V3 larvae.

**Figure 8:**
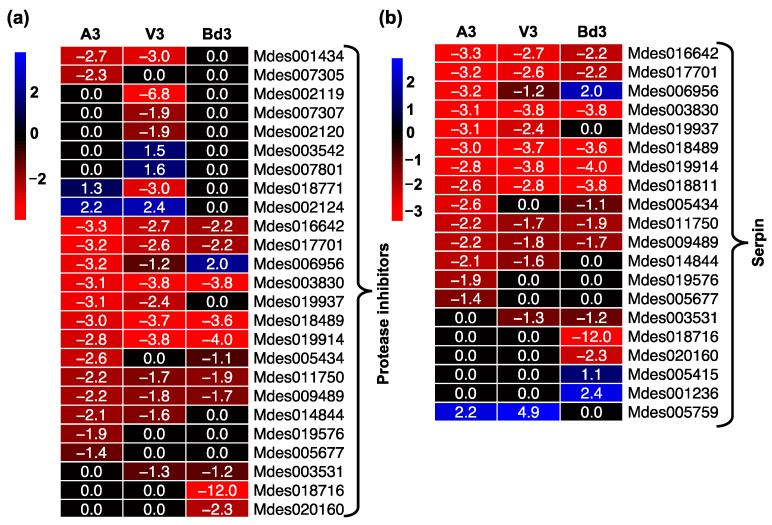
Differential expression of protease inhibitors in Hessian fly larvae 3 days after hatch (DAH). Heatmaps depict expression profiles of genes encoding protease inhibitors in avirulent (A3), virulent (V3), and Bd3 larval transcriptomes. (**a**) Differentially expressed genes encoding protease inhibitors. (**b**) Expression of genes encoding serpins, a protease inhibitor specifically targeting serine proteases. Log2 fold-change RNA-Seq values are shown within each cell of the heatmap. Blue represents up-regulated genes, and red represents the down-regulated genes, while genes not differentially expressed at a particular time-point are indicated in black.

In contrast to the expression profiles observed for proteases, most genes encoding protease inhibitors, including serpins, were mostly down-regulated in A3, V3, and Bd3 samples, with a few not differentially expressed (Figure 8, Table S7). Serpins are the largest family of serine protease inhibitors and regulate insect innate immunity by inhibiting serine proteinase cascade that triggers insect immune response [55,56]. A small fraction (15%) of the 25 genes encoding native serpins were up-regulated in Bd3, while only 3% were up-regulated in A3 and V3 larvae (Figure 8b), suggesting that in Bd3 larvae the overall protease activity may be inhibited to a greater extent than in V3, leading to lack of larval development beyond the 2nd-instar stage. 

#### 2.5.3. Regulation of Heat Shock Proteins

**Figure 9:**
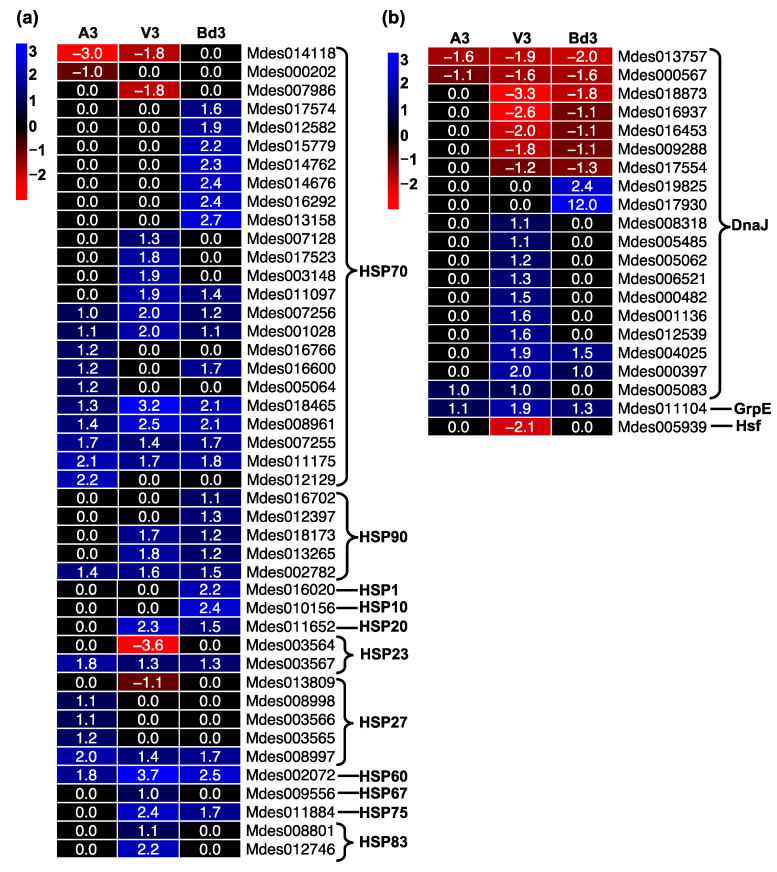
Differential expression of heat shock proteins in Hessian fly larvae 3 days after hatch (DAH). Heatmaps depict genes encoding differentially expressed heat shock proteins (HSP) in avirulent (A3), virulent (V3), and Bd3 larval transcriptomes, and grouped based on HSP families. (**a**) Expression of genes encoding HSP70, HSP90, small heat shock proteins (HSP1, HSP10, HSP20, HSP23, HSP27), HSP60, HSP67, HSP75, and HSP83. (**b**) Differentially expressed genes encoding the cochaperones DnaJ family, GrpE, and Heat shock factors (Hsf). Log2 fold-change RNA-Seq values are shown within each cell of the heatmap. Blue represents up-regulated genes, and red represents the down-regulated genes, while genes not differentially expressed at a particular time-point are indicated in black.

Upon exposure to stress, insects regulate the synthesis of heat shock proteins (HSPs) and their cochaperones DnaJ [57,58]. Based on their molecular mass, amino acid sequence, and function, HSPs are further classified into families. The major families of HSPs in insects include the small heat shock proteins (12-43 kDa), Hsp60, Hsp70, and Hsp90. In insects, these HSPs are induced to higher levels in response to external stress [57]. Our data revealed differential expression of HSPs in larvae feeding on host wheat and nonhost Bd plants (Figure 9, Table S7). The highest number of transcripts belonged to the HSP70 family, with 24 genes (Table S7). Several of these HSP70 genes were significantly up-regulated in all three samples, with 60% of up-regulated genes showing higher expression levels in A3 larvae as compared to V3 and Bd3 (Figure 9a). Except for one gene (Mdes002782), most genes encoding the HSP90 family were not significantly expressed in A3 larvae (Figure 9a). In contrast, most of the HSP90s were significantly up-regulated in V3 and Bd3 larvae (Figure 9a). One gene encoding HSP23 (Mdes003567), HSP27 (Mdes008997), and HSP60 was up-regulated in all three larvae (Figure 9a, Table S7). The transcript levels of a majority (52%) of DEGs encoding DnaJ increased in V3 but did not change (81%) in the A3 transcriptome (Figure 9b). In Bd3, while a few genes encoding DnaJ were up- or down-regulated, around 43% did not change significantly (Figure 9b). HSPs are constitutively expressed in insects but are induced to higher levels in response to stressors to maintain cell homeostasis through interaction with substrate proteins [57]. Our results suggest that HSPs are induced in A3, V3, and Bd3 larvae at varying levels to maintain a steady state of the larval cells during feeding/attempted feeding. Unlike some of the other functional groups, we observed a greater number of genes encoding certain HSPs being up-regulated in avirulent larvae, requiring further investigation into what specific roles these may be playing in avirulent versus virulent larvae. 

#### 2.5.4. Expression of Defense Proteins

**Figure 10:**
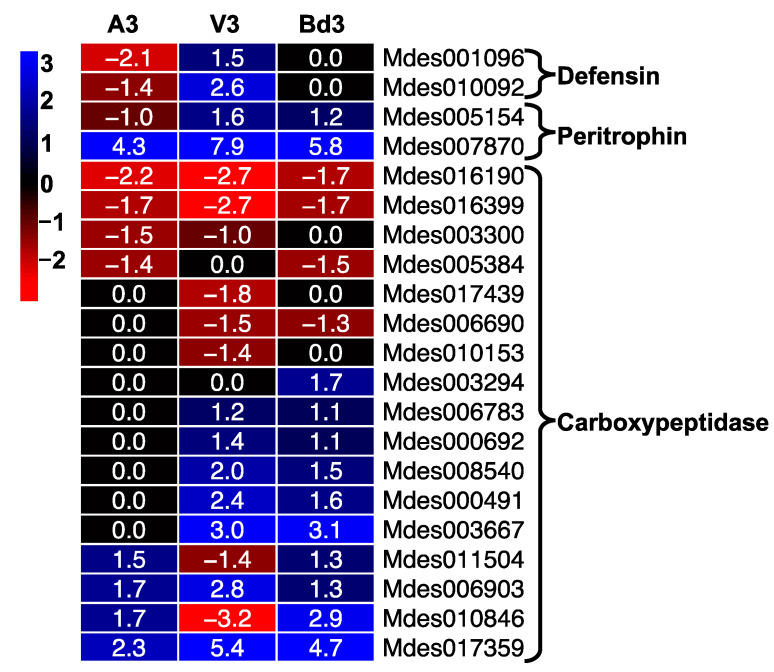
Expression of defense proteins in Hessian fly larvae 3 days after hatch (DAH). Heatmap depicts genes encoding defense-related proteins including defensins, peritrophins, and carboxypeptidases in avirulent (A3), virulent (V3), and Bd3 larval transcriptomes. Log2 fold-change RNA-Seq values are shown within each cell of the heatmap. Blue represents up-regulated genes, and red represents the down-regulated genes, while genes not differentially expressed at a particular time-point are indicated in black.

We compared the expression of genes encoding defense proteins including defensins, peritrophins, and carboxypeptidases in A3, V3, and Bd3 larval transcriptomes (Figure 10). We identified 2 genes each encoding defensin and peritrophin proteins and 17 genes encoding carboxypeptidases that were differentially expressed in larvae feeding on host wheat and nonhost Bd plants (Figure 10). The two defensin genes were down-regulated in A3 and not significantly expressed in Bd3 larvae, but were up-regulated up to 6-fold in V3 larvae (Table S7). Insect defensins are small (36–46 amino acid residues long) antimicrobial peptides, which have been proposed to play a crucial role in protection against microbial pathogens [59,60]. Damage due to Hessian fly larval feeding makes the host wheat plants vulnerable to attack by soil microorganisms [61]. Both Hessian-fly-infested wheat plants and the larvae harbor several microbes [61,62,63]. The increased expression of defensins observed in V3 larvae indicates their possible involvement in defending and protecting the larvae from any microbes from the host plant. Unlike the susceptible wheat, the larvae feeding on resistant wheat and nonhost Bd plants are unable to extensively injure the plant cell surface [25,34]. The damage is very transient and repaired promptly by the host [34] and nonhost [25] resistance mechanisms. Avirulent larvae lack microbial flora [62] and it is plausible that similar to A3 larvae, the Bd3 larvae also lack pathogenic microbial flora and therefore do not require increased expression of defensins. 

The peritrophic matrix (PM) is an important structure within the insect midgut, which aids in digestion of ingested food in gut lumen, along with protecting the midgut epithelia from invasive microorganisms and mechanical damage [64,65,66]. Ultrastructural studies clearly show that within 3 h of larval feeding on resistant wheat, the midgut microvilli are disrupted, and by 6 h are completely absent [8]. This response is unlike those observed in virulent larvae, which exhibit well-developed microvilli [8]. The peritrophins are important PM proteins that play a role in protecting the midgut from microorganisms, maintaining the PM structure, and inhibiting proteolysis [67]. Genes encoding peritrophins have been identified and cloned from several insects including *M. destructor* [67,68]. Our RNA-Seq expression data also identified two genes encoding peritrophins, with only one (Mdes007870) being highly up-regulated in all three samples (Figure 10). The expression level of this gene was as high as 231-fold in V3 and as low as 19-fold in A3, with Bd3 larvae showing intermediate levels (55-fold up-regulated) (Table S7). These results clearly show that peritrophins are crucial to larval survival. Despite high levels of this gene in A3 larvae, the other peritrophin gene, Mdes005154, is down-regulated 2-fold, and the combined levels are presumably insufficient to maintain the PM structure against the challenge of higher levels of toxic defense proteins from the resistant plants on which they are attempting feeding. 

Carboxypeptidases are exopeptidases found in insect gut that are involved in protein digestion and other physiological processes. Several of these digestive carboxypeptidases have been identified and cloned from insects [69]. In comparison with other hydrolytic enzymes, carboxypeptidases usually form a minor component of the proteases found in the insect gut [70]. It is believed that insect gut carboxypeptidases do not play a major role in digestion, and instead are involved in resistance to plant protease inhibitors [70]. A total of 17 DEGs encoding carboxypeptidases were identified in our RNA-Seq data (Figure 10). While 29% of these genes were up-regulated in A3 larvae, 65% of the genes were up-regulated in V3, and 71% genes were up-regulated in Bd3 larvae (Figure 10, Table S7). Protease inhibitors defend plants from proteases of invading insect herbivores. In fact, 13 genes encoding protease inhibitors were significantly up-regulated by 3 DAH in nonhost Bd plants [25]. Thus, it is plausible that Bd3 larvae ingest these protease inhibitors from the nonhost Bd plants and are affected in their ability to digest plant-derived nutrition, which contributes to the retardation in their growth and development beyond the 2nd-instar stage. 

#### 2.5.5. Lipases Differentially Expressed in Larvae Feeding on Host Wheat and Nonhost Bd Plants

**Figure 11:**
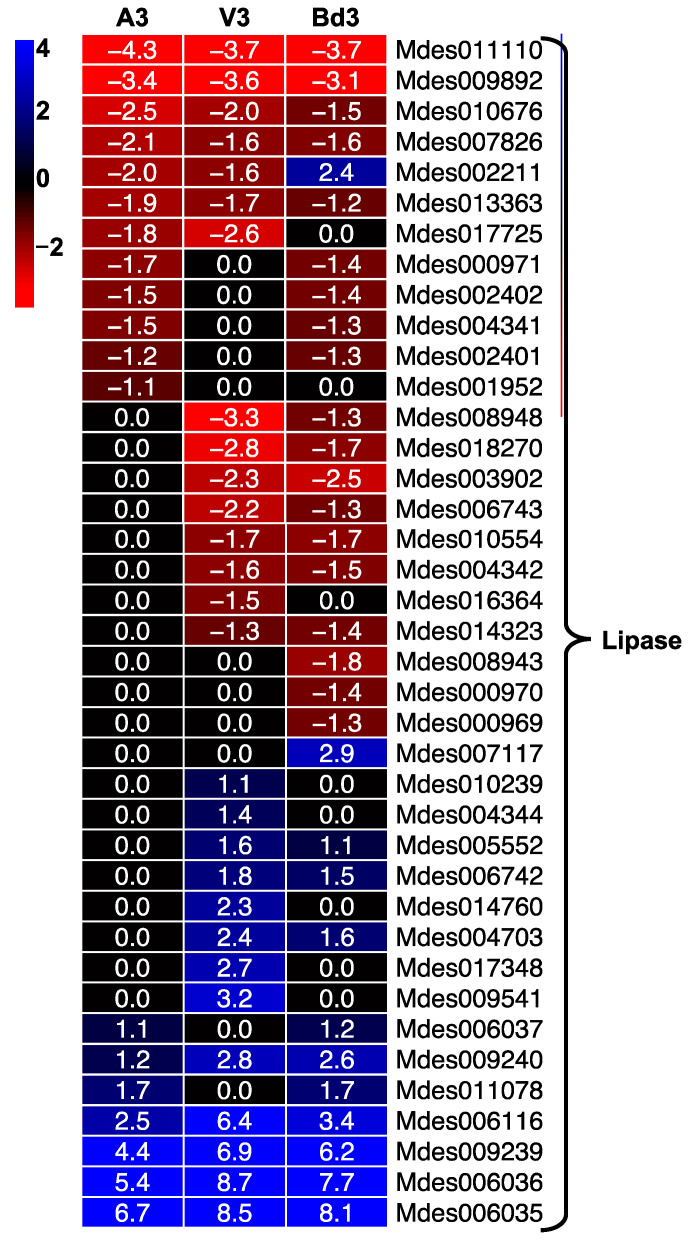
Differential expression of lipases in Hessian fly larvae 3 days after hatch (DAH). Heatmap depicts differential expression of genes encoding lipases in avirulent (A3), virulent (V3), and Bd3 larval transcriptomes. Log2 fold-change RNA-Seq values are shown within each cell of the heatmap. Blue represents up-regulated genes, and red represents the down-regulated genes, while genes not differentially expressed at a particular time-point are indicated in black.

Another functional category comprised of a large number of genes (39) and differentially expressed in the larvae feeding on host wheat and nonhost Bd plants is the lipases (Figure 11). Insect phenomena including life cycle, metamorphosis, diapause, and pheromone synthesis depend upon lipid metabolism. Lipases are enzymes that hydrolyse and regulate lipid uptake, transport, and utilization by insects [71]. In A3 larvae, 51% of the lipases were not differentially expressed, while only 18% of genes were up-regulated. In contrast, only 28% and 21% of genes were not differentially expressed in V3 and Bd3 larvae, respectively, with 31–33% of genes being up-regulated (Figure 11, Table S7). Four of the lipase-encoding genes, Mdes006116, Mdes009239, Mdes006035, and Mdes006036, showed a dramatic increase in expression levels ranging from 6- to 101-fold in A3 larvae, 82- to 428-fold in V3 larvae, and 10- to 279-fold in Bd3 larvae (Table S7). Virulent larvae feeding on susceptible host wheat show increased transcript levels of *MdesL1* (Mdes009240), a lipase identified from the salivary gland transcriptome of *M. destructor*, as compared to avirulent larvae feeding on resistant host wheat [72]. MdesL1 is proposed to be involved in extra-oral digestion and changes in host-cell permeability [72]. The increased expression of genes encoding lipases in all three types of larvae provides strong evidence for the possible involvement of this class of enzymes in regulating key metabolic processes in both avirulent and virulent larvae feeding on host wheat, as well as larvae feeding on nonhost Bd plants. 

#### 2.5.6. Genes Involved in Oxidative Stress

**Figure 12:**
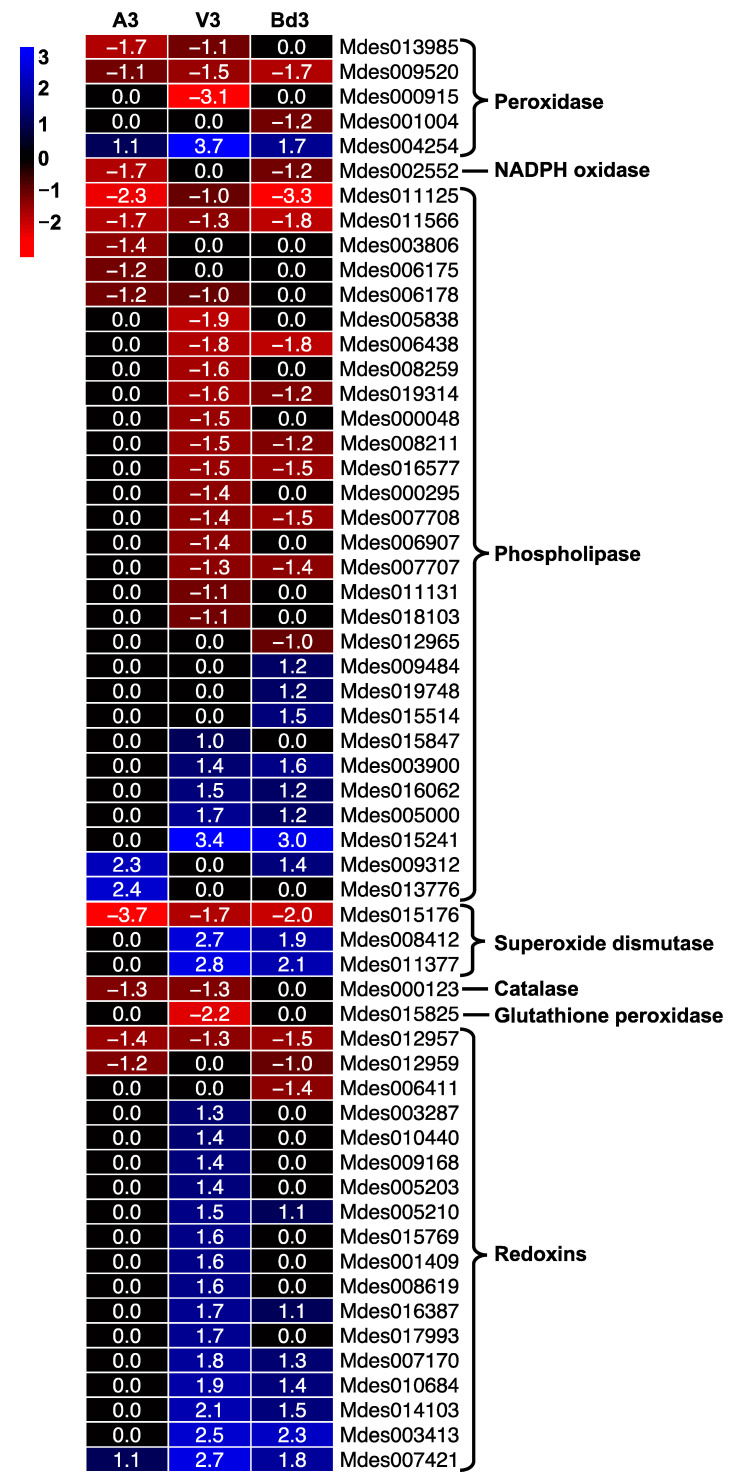
Differential expression of genes involved in oxidative stress in Hessian fly larvae 3 days after hatch (DAH). Heatmap depicts the expression profiles of genes encoding enzymes involved in reactive oxygen species (ROS) generation (peroxidases, NADPH-oxidase, phospholipases) and ROS scavenging (superoxide dismutases, catalase, glutathione peroxidase, redoxins) in avirulent (A3), virulent (V3), and Bd3 larval transcriptomes. Log2 fold-change RNA-Seq values are shown within each cell of the heatmap. Blue represents up-regulated genes, and red represents the down-regulated genes, while genes not differentially expressed at a particular time-point are indicated in black.

Phytophagous insects are constantly challenged with reactive oxygen species (ROS) radicals that are generated both exogenously and endogenously by defense allelochemicals produced by the host plant on which they are feeding or as a response to stress within the insects [73,74]. We identified genes involved in oxidative stress in larvae feeding on host and nonhost plants. We identified DEGs involved in both ROS generation and scavenging processes (Figure 12). The ROS-generating genes included peroxidases, NADPH oxidase, and phospholipases that were differentially expressed (Figure 12). While NADPH-oxidase was down-regulated in both A3 and Bd3, it was not differentially expressed in V3 larvae (Figure 12). Of the five peroxidase DEGs identified, only one (Mdes004254) was up-regulated in all three larval samples. Twenty-nine DEGs encoding phospholipases were identified. Of these, 76% were not differentially expressed in A3 larvae and only two were up-regulated, interestingly, at higher levels than in V3 and Bd3 (Figure 12). In V3 and Bd3 larvae, five and eight genes were up-regulated, respectively, with the transcript abundance of Bd3 larvae for these genes being lower than in the V3 larvae (Table S7). Hessian flies have a suite of ROS-scavenging enzymes as part of antioxidant defense [75]. Increased levels of mRNA encoding two each of glutathione peroxidases and catalases were observed in both virulent and avirulent larvae feeding on host resistant and susceptible wheat, respectively [75]. Transcripts for genes encoding superoxide dismutase only increased in virulent larvae but were not differentially expressed in the avirulent larvae [75]. Similar to this previously documented data, our RNA-Seq data also revealed increased transcript levels for two of the three superoxide dismutases in V3 and Bd3 larvae, with no differential expression in A3 larvae (Figure 12, Table S7). However, the RNA-Seq expression profile for the gene encoding catalase and glutathione peroxidase did not match the expression profile documented previously in Biotype L larvae [75]. Both genes were either down-regulated or not differentially expressed in all three larval types (Figure 12). In the previous study, the expression values for both catalases and glutathione peroxidases were generated using ubiquitin (Mdes005097) as the endogenous reference. Our RNA-Seq data reveals that since ubiquitin is differentially expressed in A3 (2-fold up-regulated) as well as V3 larvae (7.4-fold up-regulated), the expression values reported by Mittapalli et al. [75] are over-estimated. Most of the redoxins identified were not differentially expressed in A3 larvae, while most of them were up-regulated in V3 larvae (Figure 12). Bd3 larvae had fewer DEGs for redoxin, which also showed lower transcript abundance compared to V3 larvae. These results clearly indicate that A3 larvae are unable to protect themselves from any oxidative damage caused by ROS generated by the host plants and die, while the virulent V3 larvae are able to scavenge ROS and complete their growth and development. On the other hand, the Bd3 larvae generate more endogenous ROS and scavenge less exogenous ROS from the Bd plant, possibly causing oxidative damage, which may be contributing to their retarded development and ultimately death after several days. 

#### 2.5.7. Modulation of Nonessential Amino Acid Biosynthetic Pathway (NAABP)

**Figure 13:**
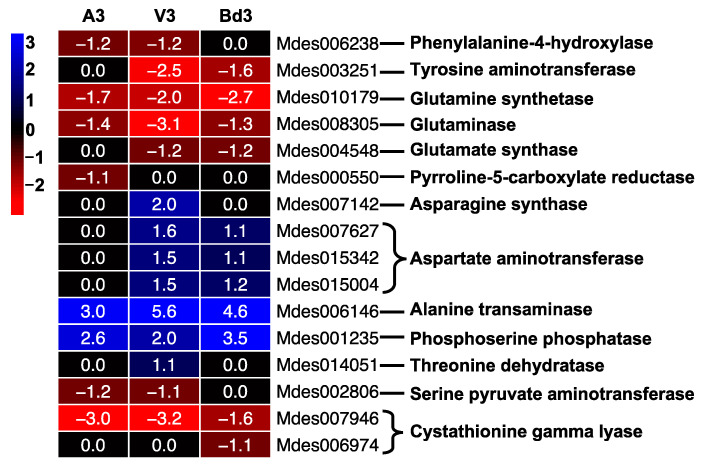
Differential expression of genes from the nonessential amino acid biosynthetic pathway (NAABP) in Hessian fly larvae 3 days after hatch (DAH). Heatmap depicts differential expression trends of genes that are part of synthesis of nonessential amino acids in avirulent (A3), virulent (V3), and Bd3 larval transcriptomes. Log2 fold-change RNA-Seq values are shown within each cell of the heatmap. Blue represents up-regulated genes, and red represents the down-regulated genes, while genes not differentially expressed at a particular time-point are indicated in black.

The successful establishment of larval feeding sites is key to a compatible interaction between wheat and Hessian fly, leading to plant susceptibility. Virulent larvae reprogram the susceptible host plant’s physiology, making the plant a nutrient sink enriched with essential and nonessential amino acids [9,13,14]. We previously demonstrated that besides exploiting the host wheat to obtain essential amino acids for nutritional benefits, Hessian flies also utilize the plant-derived nonessential amino acids and complement those with de novo synthesis of nonessential amino acids to overcome any deficiencies, after nutritive tissue formation [15]. Our RNA-Seq expression data (Figure 13) was consistent with the results documented previously for NAABP genes. Transcript abundance for genes involved in the biosynthesis of tyrosine, glutamine, glutamic acid, and proline declined in feeding instars on both host wheat and nonhost Bd plants as compared to the neonates (Figure 13, Table S7). However, transcripts accumulated for alanine and serine synthesis genes in all the larval samples (Figure 13), resembling the expression profile reported previously for these genes [15]. The Hessian fly modulates the NAABP depending upon the larval dietary needs and ability to utilize plant-derived nonessential amino acids in response to induced resistance and susceptibility and nonhost resistance. 

#### 2.5.8. Regulation of Tricarboxylic Acid (TCA) Pathway

**Figure 14:**
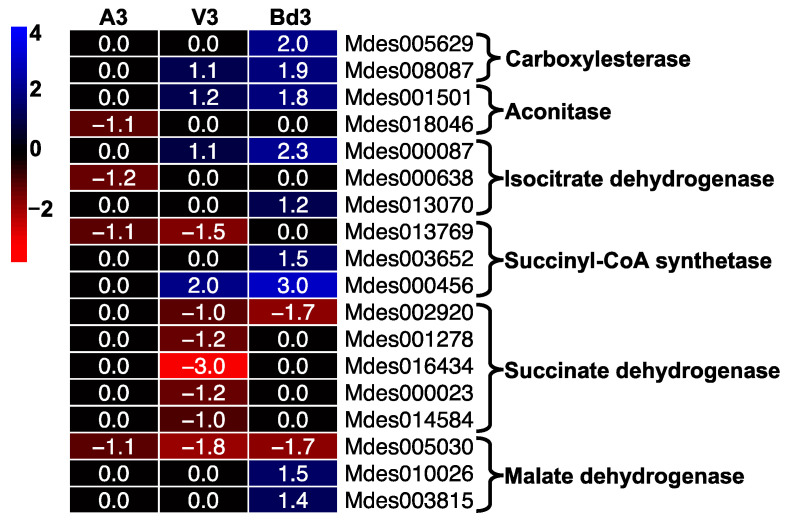
Regulation of genes from the tricarboxylic acid (TCA) pathway in Hessian fly larvae 3 days after hatch (DAH). Heatmap depicts changes in transcript levels of genes encoding enzymes of the TCA pathway in avirulent (A3), virulent (V3), and Bd3 larval transcriptomes. Log2 fold-change RNA-Seq values are shown within each cell of the heatmap. Blue represents up-regulated genes, and red represents the down-regulated genes, while genes not differentially expressed at a particular time-point are indicated in black.

The TCA pathway is extremely important for energy metabolism in insects. DEGs encoding enzymes in the TCA pathway were represented in our RNA-Seq data by 18 genes (Figure 14). These results are consistent with the expression profiles of genes involved in citric acid cycle and energy metabolism in Hessian fly Biotype GP reported previously [33]. A higher percentage of genes from this pathway were differentially expressed in virulent larvae feeding on the susceptible wheat line Newton as compared to avirulent larvae feeding on the resistant wheat line Molly [33]. Except for genes encoding succinate dehydrogenase and malate dehydrogenase, other genes including citrate synthase, aconitase, isocitrate dehydrogenase, and succinyl-CoA synthetase were significantly up-regulated in V3 larvae, with transcripts accumulating between 2 and 8 folds (Table S7). In contrast, most genes from this pathway were not differentially expressed in A3 larvae, except for a few that were down-regulated (Figure 14). Insects have to expend energy regularly for various metabolic processes. Significant changes to expression of TCA pathway genes have been reported in the internal metabolism of insects during metamorphosis [76]. The significant increase in expression of TCA cycle genes in V3 larvae clearly indicates the up-regulation of energy metabolism during larval growth and development on the susceptible host wheat. Lack of expression of these genes in A3 larvae can be correlated with the larvae dying within 4–5 DAH. The Bd3 larvae had higher transcript abundance for most of the TCA pathway genes compared to V3 (Figure 14), suggesting a higher energy requirement for these larvae as they attempt to feed and prolong their survival on nonhost Bd plants. 

#### 2.5.9. Differential Expression of Genes Encoding Secreted Salivary Gland Proteins

**Figure 15:**
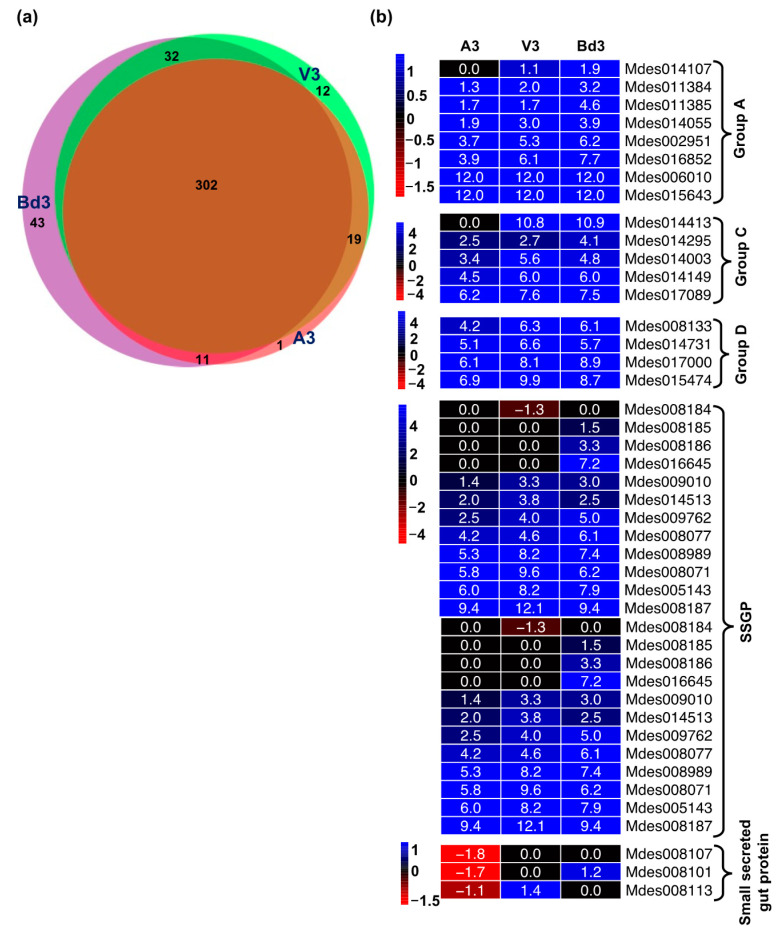
Differential expression of Secreted Salivary Gland Proteins (SSGPs) in Hessian fly larvae 3 days after hatch (DAH). (**a**) Venn diagram showing number of differentially expressed genes encoding SSGPs that are shared and uniquely expressed between A3, V3, and Bd3 larval transcriptomes. (**b**) Heatmaps depicting transcriptional changes in genes representing various SSGP families in A3, V3, and Bd3 larvae. Log2 fold-change RNA-Seq values are shown within each cell of the heatmap. Blue represents up-regulated genes, and red represents the down-regulated genes, while genes not differentially expressed at a particular time-point are indicated in black.

It is well documented that many insects use an effector-based strategy to alter the host plant’s physiology [77,78]. The Hessian fly larvae inject large numbers of secreted salivary gland proteins (SSGPs) belonging to different families into the host plant during feeding to manipulate the wheat plant’s physiological processes [79,80,81,82,83,84]. Our RNA-Seq study identified a large number of SSGPs that were differentially expressed in A3, V3, and Bd3 larvae feeding on host and nonhost Bd plants (Table S7). The Venn diagram depicting shared and unique genes revealed a large number of genes (302) encoding SSGPs that were common between A3, V3, and Bd3 larvae (Figure 15a). These SSGPs belonged to several groups including A, B, C, D, E, and F [81], along with small secreted gut proteins and others (Figure 15b, Table S7) that have been cloned and characterized in detail. The largest number of SSGPs belonged to the groups B and F (Table S7). In both of these groups, most DEGs were significantly up-regulated in A3, V3, and Bd3 larvae. Similar expression trends were also observed for SSGPs from groups A, C, and D, and other SSGPs with significant up-regulation in all three larval samples (Figure 15b). Most SSGPs showed very high transcript abundance, with several genes showing as high as 4000-fold up-regulation (Table S7). Interestingly, the small secreted gut proteins were down-regulated in A3, and one each upregulated in V3 and Bd3 larvae (Figure 15b). The large number of SSGPs with very high mRNA abundance clearly point towards a key significant role played by these effector proteins during larval feeding. It appears that while these SSGPs in virulent larvae are able to alter the host plant physiology and induce susceptibility [16], the *H*-gene-mediated resistance in host wheat and nonhost resistance in Bd plants prevents these effector SSGPs from A3 and Bd3 larvae, respectively, from triggering major permanent physiological changes in the plants. 

## 3. Materials and Methods

### 3.1. Insect and Plant Material

The Hessian fly (*Mayetiola destructor*) Biotype L stock used in this study was maintained in cold (4 °C) storage in a diapause stage as per Sosa and Gallun [85]. Two near-isogenic wheat (*Triticum aestivum*) lines “Iris” (harboring the *H9* resistance gene) and “Newton” (lacking the resistance gene) were used as resistant and susceptible host plants to Biotype L Hessian fly, respectively. *Brachypodium distachyon* (Bd) seeds of line Bd21 were used as nonhost plant material to Hessian fly in this study. 

### 3.2. Plant Growth and Infestation

Resistant and susceptible wheat cultivars were grown as described previously [15]. Briefly, seeds for each wheat line (Iris and Newton) were grown in 4-inch pots (12 seeds per pot) containing Promix Professional soil (Premier Horticulture Inc., Quakertown, PA, USA). The plants were grown in a growth chamber (Conviron, Controlled Environments Limited, Winnipeg, Manitoba, Canada) set at 18 °C, with a 16 h/8 h (light/dark) photoperiod (irradiance between 980 and 1470 μmol m^−2^ s^−1^) and 60% relative humidity. To infest the plants, at 1-leaf stage, pots were covered with vented plastic cups, following which 3 female and 2 male Hessian flies were introduced within each pot. Bd nonhost plants were grown as described in Hargarten et al. [32]. Ten seeds were planted in each 4-inch pot containing equal volume (50:50 mix) of growing medium:vermiculite (Perlite Vermiculite Packaging Industries, North Bloomfield, OH, USA) and Farfard professional potting mix (Conrad Farfard Inc., Agawam, MA, USA) and grown in a growth chamber set at 18 °C, with 24 h photoperiod and 60% relative humidity. Bd plants were infested at the 3-leaf stage with 10 female and 2 male Biotype L flies (per pot) as described in Hargarten et al. [32]. 

### 3.3. Insect Tissue Collections

The avirulent 1st-instar Biotype L larvae feeding on resistant Iris wheat were collected on 1 (A1) and 3 (A3) days after egg hatch (DAH). Similar collections were done for virulent 1st-instar Biotype L larvae on 1 (V1) and 3 (V3) DAH feeding on susceptible Newton wheat line. First- and second-instar Biotype L larvae feeding on Bd plants were collected on 3 (Bd3) and 9 (Bd9) DAH, respectively. Neonate (newly hatched) Biotype L control larvae that had not fed on the plants were collected from infested Newton (N0) and Bd (BdN) plants. All larval collections from host wheat and nonhost Bd plants were done as described in Subramanyam et al. [15], flash-frozen in liquid nitrogen, and stored at −80 °C. Larval tissues were collected from three biological replicates from 30 plants per time point per replicate. Each biological replicate per time point was represented by around 500–550 larvae pooled together from the 30 plants. 

### 3.4. RNA Isolation and Quantification

Total RNA was extracted from the frozen larvae collected from host and nonhost plants, using TRIzol reagent according to the manufacturer’s protocol (Life Technologies Corporation, Carlsbad, CA, USA). RNA concentration of each sample was determined using Nanodrop ND-1000 spectrophotometer (NanoDrop Technologies, Wilmington, DE, USA). RNA quality was assessed using Agilent 2100 Bioanalyzer (Agilent Technologies, Santa Clara, CA, USA), and only the RNA samples with integrity number (RIN) ≥ 8 were used for library preparation.

### 3.5. Illumina RNA-Sequencing and Data Processing

RNA-Seq library construction and sequencing was performed at the Purdue University Genomics Core Facility. The cDNA libraries were prepared using the TruSeq RNA Sample Prep Kit (Illumina Inc., San Diego, CA, USA) as per manufacturer’s instructions. The libraries were then barcoded, pooled, and sequenced on HiSeq2500 instrument (Illumina Inc.) using paired end sequencing (100 bp and 150 bp reads for libraries made from larvae on wheat and Bd plants, respectively). The quality of the Illumina sequence reads for all samples was assessed using FastQC (v.0.11.7; https://www.bioinformatics.babraham.ac.uk/projects/fastqc/ accessed on 12 December 2018). Quality trimming was performed using FASTX-Toolkit (v.0.01.4; http://hannonlab.cshl.edu/fastx_toolkit/ accessed on 12 December 2018) to remove bases with Phred33 score of less than 30, and the resulting reads with lengths of at least 50 bases were retained. The quality trimmed reads were mapped against the *Mayetiola destructor* reference genome (https://www.ncbi.nlm.nih.gov/nuccore/307816351 accessed on 20 December 2018). Mapping results and reference genome annotations were used as input for HTSeq package (v.0.7.0) [86] to obtain the read counts for each gene feature for each biological replicate. Counts from all replicates were merged to generate a read count matrix. In order to visualize the variation within and between samples, Principal Component Analysis (PCA) plots were generated using DESeq2 with larval samples feeding on host wheat and nonhost Bd. 

### 3.6. Identification and Analysis of Differentially Expressed Genes

Pairwise comparisons were carried out between A1, A3, V1, and V3 samples vs. the neonates feeding on wheat (N0). Similarly, Bd3 and Bd9 samples were compared to neonates feeding on Bd plants (BdN). Gene expression analysis between treatment and control was carried out using ‘R’ Bioconductor (v.3.4.2; http://www.r-project.org/ accessed on 7 January 2019), using three methods, edgeR (v.3.20.9 [87]), DESeq2 (v.1.18.1 [88]), and Cufflinks (v.2.2.1 [89]), to identify differentially expressed genes (DEGs). The criteria used to extract DEGs were False Discovery Rate (FDR) adjusted *p*-value < 0.05 with log2 expression >1 or <−1, which translated to linear fold change (FC) >2 or <−2. DEGs that were detected in two or more methods were identified, and the mean log2 FC value was generated for each gene. For DEGs from Cufflink results which had log2 FC as “inf” or “-inf”, the values were converted to 12 or −12, respectively. To identify functionally annotated genes, the *M. destructor* reference protein sequence was used as input to perform blastx (blast standalone v.2.5.0 [90]) against the nonredundant (nr) protein database, and genomes of *Aedes aegypti* (https://metazoa.ensembl.org/Aedes_aegypti_lvpagwg/Info/Index accessed on 1 March 2021), *Anopheles gambiae* (https://metazoa.ensembl.org/Anopheles_gambiae/Info/Index accessed on 1 March 2021), and *Drosophila melanogaster* (https://metazoa.ensembl.org/Drosophila_melanogaster/Info/Index accessed on 1 March 2021) to identify the top blast hit using *E*-value cutoff of 10^−3^. Venn diagrams with DEGs were created using DeepVenn (https://www.deepvenn.com/ [91] accessed on 1 September 2021). Clust (v.1.12.0) was run on the DEGs using Z-score normalization [92]. Gene Ontology (GO) (BP: biological process, CC: cellular component, MF: molecular function) and KEGG pathway enrichment analysis of DEGs identified from larval samples A3, V3, and Bd3 were performed using clusterProfiler (v.3.14.0 [93]). Pheatmap (v.1.0.12) was used to draw heatmaps with DEGs using ‘R’ Bioconductor.

### 3.7. Validation of RNA-Seq Gene Expression

To validate the RNA-Seq expression data, the expression profiles of 10 representative DEGs were confirmed by quantitative reverse transcription-polymerase chain reaction (qRT-PCR) using LightCycler 480 Instrument II (Roche Diagnostics Corporation, Indianapolis, IN, USA) as described previously [16]. Transcript-specific primers (Table S8) were designed with Primer Express 3.0 software from Applied Biosystems (ABI, Foster City, CA, USA). qRT-PCR was performed in triplicate for all three biological replicates. Hessian fly 18S ribosomal RNA was included in qRT-PCR as the endogenous control for larval samples and gene-specific forward (5′ CACGCGCGCTACAATGAA-3′) and reverse (5′-ACGGTTTACCCGAGCCTTTAG-3′) primers were designed from GenBank accession number KC177284. Relative Standard Curve method (ABI User Bulletin 2, ABI PRISM 7700 Sequence Detection System) was used for quantification of transcript abundance as described previously [15]. Fold change of expression in Hessian fly larval samples was calculated as the ratio of transcript levels in larvae that have started feeding after egg hatch to the neonates that are assumed to be non-feeding. 

## 4. Conclusions

Comparative transcriptome analysis of Hessian fly larvae residing on resistant and susceptible host wheat plants and nonhost Bd plants revealed overall similar molecular responses between V3 and Bd3 larvae that were distinct from those observed in A3 larvae. Differentially expressed genes involved in energy and amino acid metabolism, ROS pathway, proteases, lipases, and detoxification were significantly up-regulated in both V3 and Bd3 larvae, and are beneficial for the growth and development of the larvae. Genes from most of these pathways were either down-regulated or not differentially expressed in A3 larvae. A large number of SSGPs were significantly up-regulated in all three larval samples. Despite sharing common molecular responses, Bd3 larvae are unable to induce susceptibility in nonhost plants, unlike the V3 larvae. The primary factor responsible for this may be the relatively decreased transcriptional abundance of the DEGs as compared to the V3 larvae, which allows some of the Bd larvae to form developmentally delayed 2nd-instars with prolonged larval survival, ultimately yielding to the nonhost resistance defense mechanisms and dying. In contrast, in susceptible host wheat, due to the lack of corresponding *H*-gene-mediated defense responses, the virulent larvae are able to successfully establish permanent feeding sites, alter the host plant physiology, and complete their development. In resistant wheat, the *H*-gene-mediated early defense induces resistance, which is reflected in a lack of dynamic transcriptional changes in the avirulent larvae during attempted feeding. Understanding the insect global molecular responses and adaptation strategies will be crucial in developing effective management strategies to control these insect pests. 

## Data Availability

Illumina sequencing reads from these transcriptomes have been submitted to the NCBI Sequence Read Archive (PRJNA766660).

## Supplementary Materials

Supplementary materials can be found at https://www.mdpi.com/article/10.3390/ijms222111498/s1.

## Abbreviations

DEG	Differentially expressed gene
SSGP	Secreted salivary gland protein
HSP	Heat shock protein
PI	Protease inhibitor
TCA	Tricarboxylic acid cycle
NAABP	Nonessential amino acid biosynthetic pathway
ROS	Reactive oxygen species
UGT	UDP-glycosyltransferase
GST	Glutathione-S-transferase
CYP450	Cytochrome P450
PM	Peritrophic matrix
