# Supplementary material for: Comparative Hessian Fly Larval Transcriptomics Provides Novel Insight into Host and Nonhost Resistance
Source: Int J Mol Sci. 2021 Oct 25;22(21):11498. doi: 10.3390/ijms222111498 (PMC8583952; doi:10.3390/ijms222111498)
Supplement: Supplementary file 1 [file ijms-22-11498-s001.zip › Supplementary-Figures.pdf]

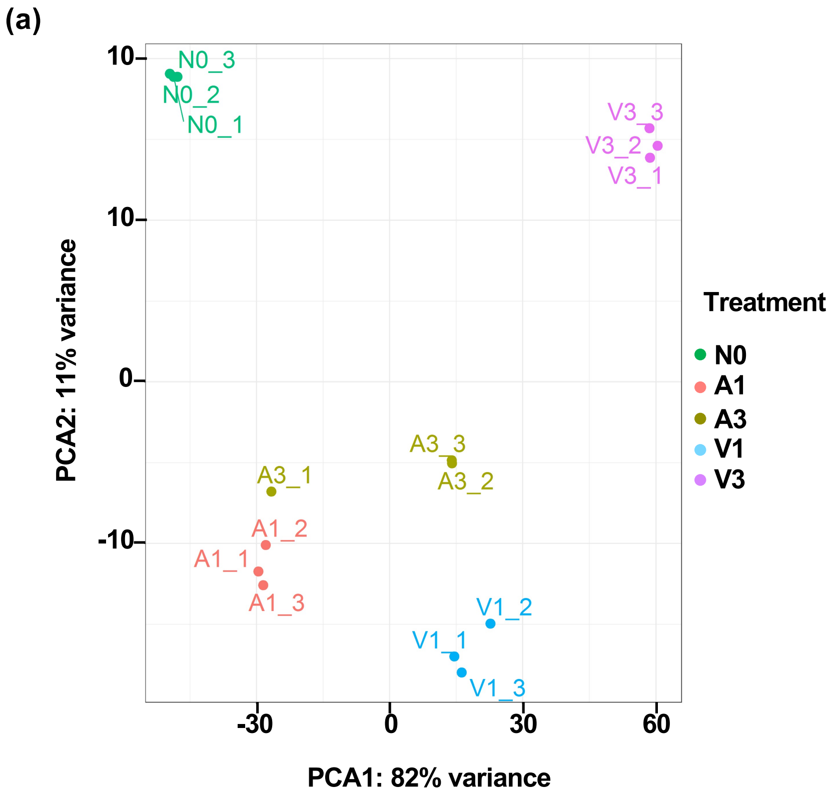

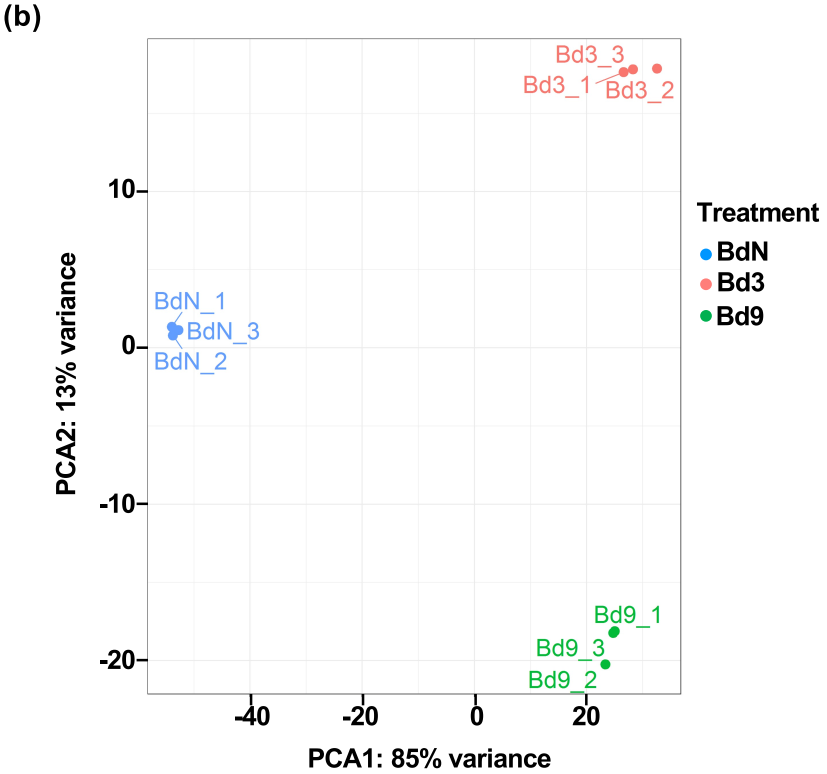

Figure S1. Principal component analysis (PCA) of all larval samples. (**a**) PCA plot for neonate (N0) and avirulent (A1 and A3) and virulent (V1 and V3) larval samples collected from host wheat at 1 and 3 days after hatch (DAH); (**b**) PCA plot for neonates (BdN) and larvae collected from nonhost Bd plants at 3 (Bd3) and 9 (Bd9) DAH.

(a)

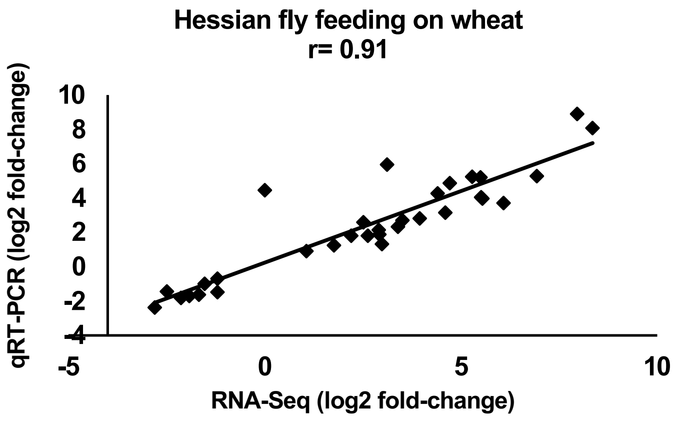

(b)

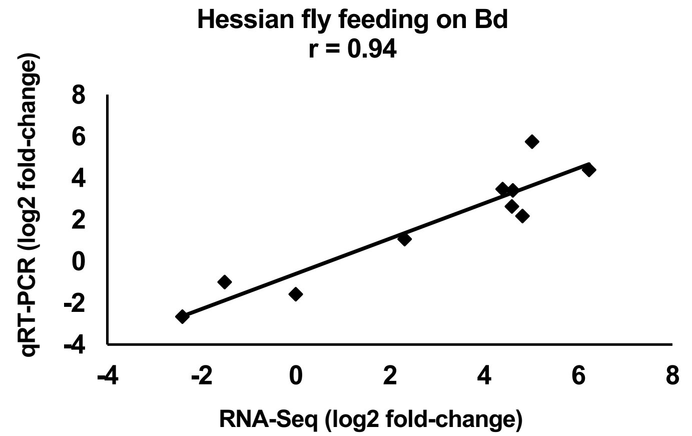

Figure S2. Correlation between RNA-Seq and qRT-PCR expression data. Pearson correlation coefficient was used to determine the similarity in gene expression pattern between RNA-Seq and qRT-PCR for 10 representative differentially expressed genes (DEG). All expression data were normalized in log2 scale.

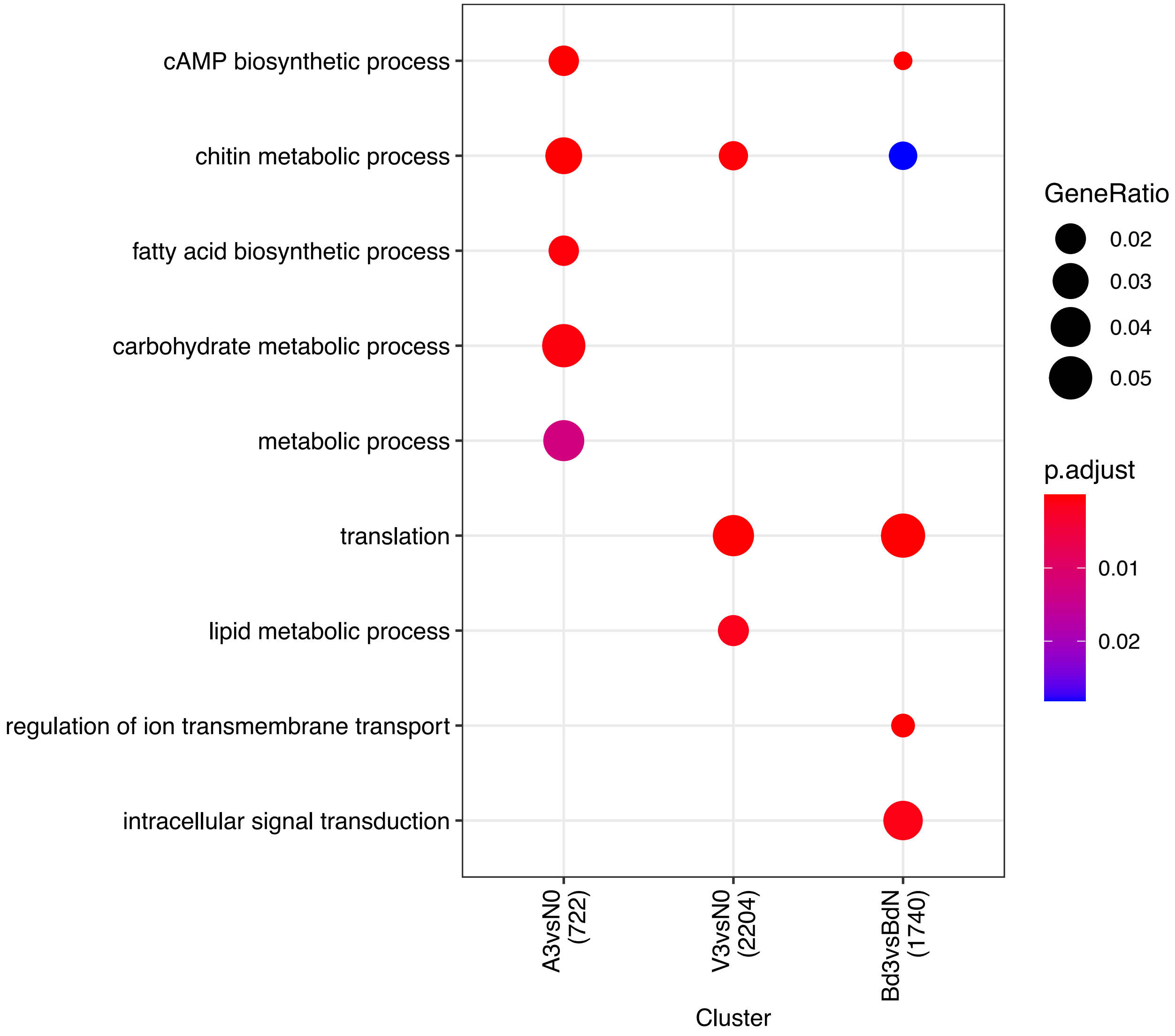

Figure S3. GO enrichment analysis for genes in biological processes. Dot plot shows differentially expressed GO terms (p<0.05) representing biological processes in avirulent (A3), virulent (V3) and Bd3 larval transcriptomes. The size of the dot is based on gene count enriched in the pathway, and the color of the dot shows the pathway enrichment significance.

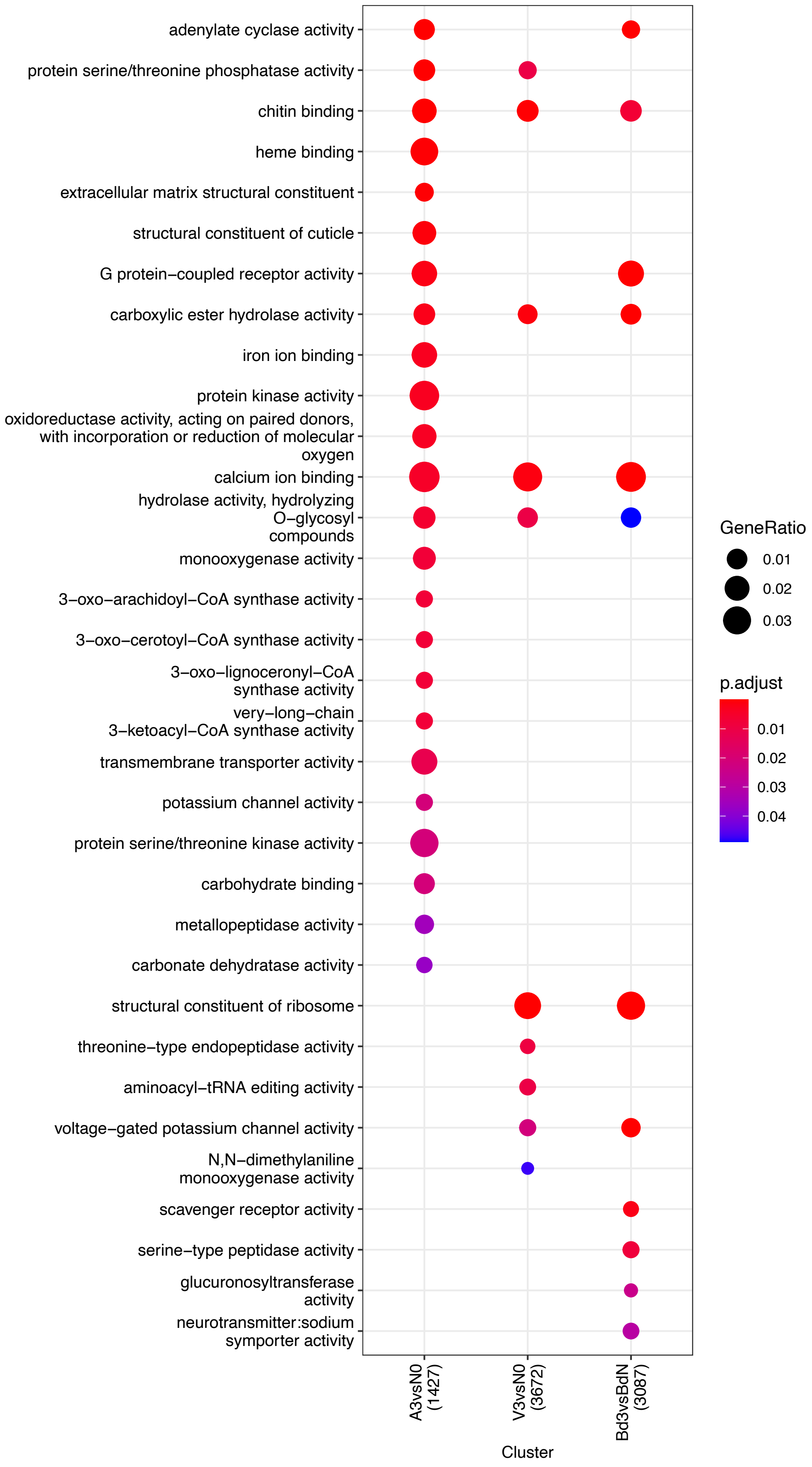

Figure S4. GO enrichment analysis for genes in molecular function. Dot plot shows differentially expressed GO terms (p<0.05) representing molecular function in avirulent (A3), virulent (V3) and Bd3 larval transcriptomes. The size of the dot is based on gene count enriched in the pathway, and the color of the dot shows the pathway enrichment significance.

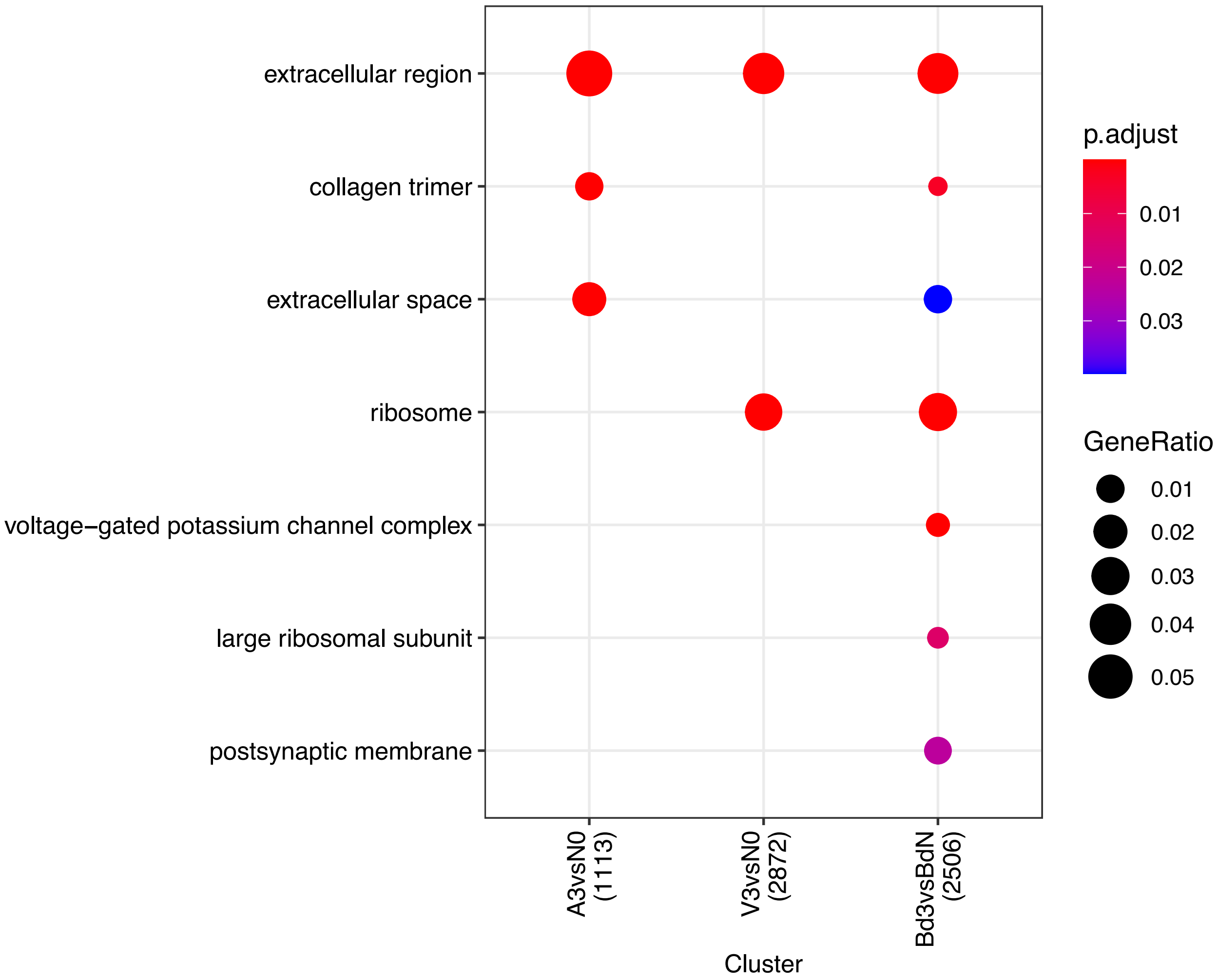

Figure S5. GO enrichment analysis for cellular component genes. Dot plot shows differentially expressed GO terms (p <0.05) representing cellular component in avirulent (A3), virulent (V3) and Bd3 larval transcriptomes. The size of the dot is based on gene count enriched in the pathway, and the color of the dot shows the pathway enrichment significance.

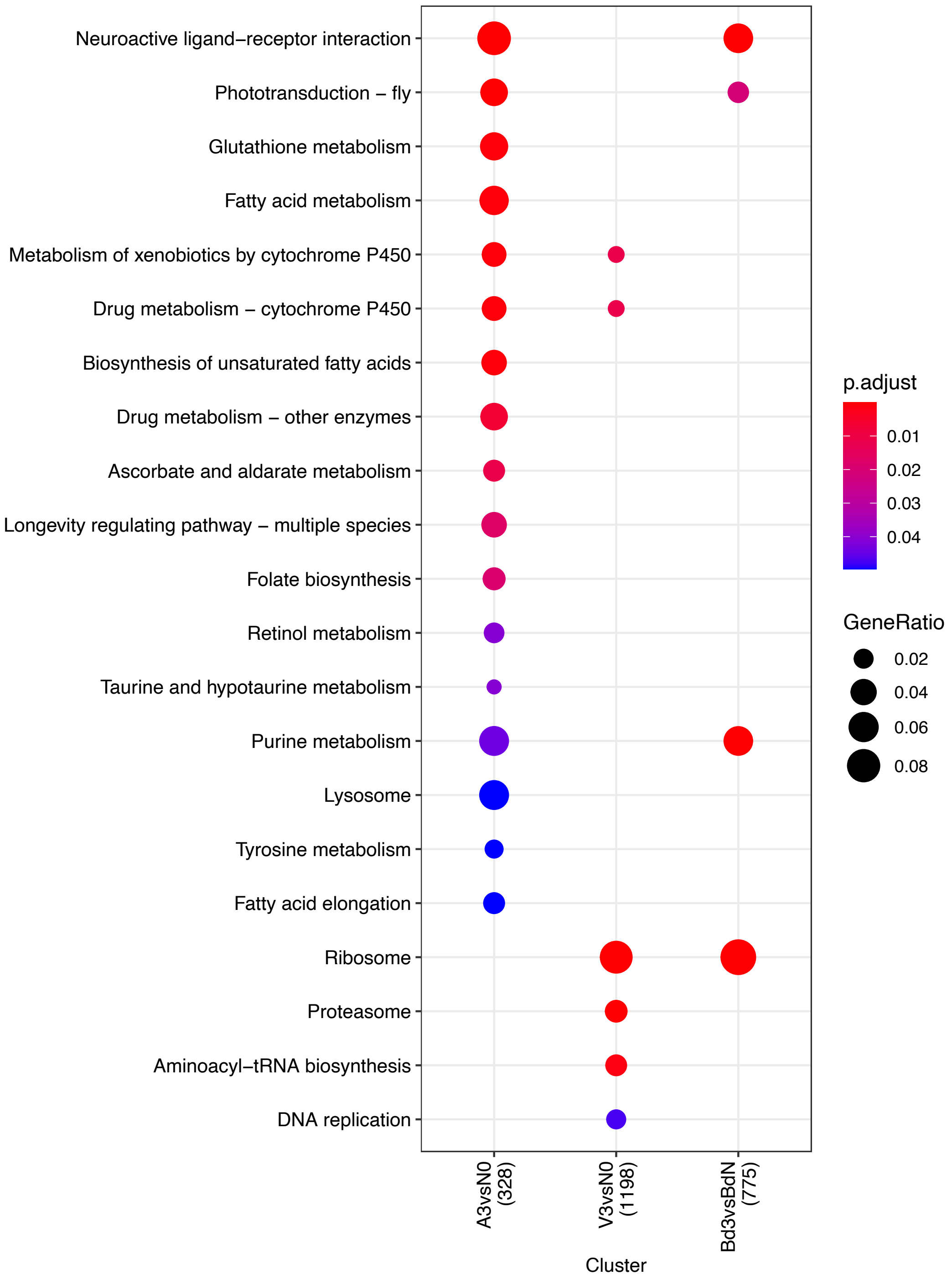

Figure S6. KEGG enrichment analysis. Dot plot shows differentially expressed KEGG pathways ($p < 0.05$) in avirulent (A3), virulent (V3) and Bd3 larval transcriptomes. The size of the dot is based on gene count enriched in the pathway, and the color of the dot shows the pathway enrichment significance.
