# Supplementary material for: Comparative Hessian Fly Larval Transcriptomics Provides Novel Insight into Host and Nonhost Resistance
Source: Int J Mol Sci. 2021 Oct 25;22(21):11498. doi: 10.3390/ijms222111498 (PMC8583952; doi:10.3390/ijms222111498)
Supplement: Supplementary file 1 [file ijms-22-11498-s001.zip › TableS1.pdf]

Table S1. Summary of RNA-Seq reads generated using the *Mayetiola destructor* reference genome

Sample Name*	Total Reads	Clean Reads	Clean Data Rate (%)	Total Reads Mapped	Mapping Rate (%)	Uniquely Mapped Reads	Uniquely Mapped Rate (%)
Larvae feeding on host wheat plants							
N0-1	95,310,580	93,508,694	98.11	69,381,946	74.19	55,353,414	79.78
N0-2	76,621,244	75,067,556	97.97	55,977,177	74.57	46,963,834	83.90
N0-3	82,143,168	80433,838	97.92	59,205,105	73.61	49,638,720	83.84
A1-1	80,074,418	78,491,330	98.02	58,669,278	74.75	49,821,852	84.92
A1-2	91,999,020	90,246,234	98.09	67,698,265	75.02	57,826,042	85.42
A1-3	100,468,304	98,695,632	98.24	74,420,097	75.40	63,609,152	85.47
A3-1	77,050,978	75,683,174	98.22	57,817,807	76.39	49,720,566	86.00
A3-2	88,243,092	86,720,082	98.27	66,821,538	77.05	57,332,206	85.80
A3-3	88,868,684	87,359,052	98.30	67,432,058	77.18	57,782,818	85.69
V1-1	91,870,360	90,292,692	98.28	70,152,652	77.69	60,807,860	86.68
V1-2	75,583,742	74,156,872	98.11	57,479,345	77.51	49,891,442	86.80
V1-3	84,055,120	82,676,270	98.36	64,096,957	77.52	55,373,562	86.39
V3-1	83,480,172	82,087,078	98.33	66,006,996	80.41	56,325,402	85.33
V3-2	95,182,932	93,559,854	98.29	75,212,615	80.38	64,917,600	86.31
V3-3	71,958,678	70,664,708	98.20	56,965,238	80.61	49,179,358	86.33
Larvae feeding on nonhost Bd plants							
BdN-1	271,413,320	271,206,148	99.92	232,084,962	85.64	143,303,452	61.75
BdN-2	248,268,344	246,066,945	99.92	211,141,016	85.87	138,861,978	65.77
BdN-3	232,545,838	232,363,645	99.92	189,719,248	81.7	125,715,384	66.26
Bd3-1	263,209,332	263,036,089	99.93	224,648,422	85.46	164,376,122	73.17
Bd3-2	249,027,000	248,823,334	99.92	220,840,202	88.83	152,940,942	69.25
Bd3-3	256,090,720	255,918,562	99.93	216,884,502	84.81	163,322,018	75.30
Bd9-1	233,947,630	233,781,987	99.93	196,867,946	84.27	134,898,956	68.52
Bd9-2	202,824,028	202,692,167	99.93	167,900,950	82.88	109,357,544	65.13
Bd9-3	194,618,920	194,481,146	99.93	162,830,874	83.78	112,358,084	69.00

*The numbers 1, 2 and 3 at the end of the sample name represent the three biological replicates.
