# Supplementary material for: Comparative Hessian Fly Larval Transcriptomics Provides Novel Insight into Host and Nonhost Resistance
Source: Int J Mol Sci. 2021 Oct 25;22(21):11498. doi: 10.3390/ijms222111498 (PMC8583952; doi:10.3390/ijms222111498)
Supplement: Supplementary file 1 [file ijms-22-11498-s001.zip › TableS2.pdf]

Table S2. Validation of RNA-Seq expression data by qRT-PCR

	RNA-seq					qRT-PCR				
**Gene_id**	**A1**	**V1**	**A3**	**V3**	**Bd3**	**A1**	**V1**	**A3**	**V3**	**Bd3**
Mdes018462	-	4.6	2.1	10.5	5.0	-	3.5	1.9	5.0	2.1
Mdes018785	6.2	24.2	15.4	68.2	28.2	3.5	8.9	7.1	13.0	4.5
Mdes009247	3.4	26.4	5.7	39.2	21.0	2.4	29.6	6.0	38.1	11.2
Mdes009774	-2.9	-4.8	-2.5	-7.0	-2.8	-	-	-	-5.1	-2.0
Mdes009239	11.4	45.2	21.2	122.0	74.9	6.5	37.2	19.5	38.9	20.9
Mdes010266	-2.9	-3.8	-5.6	-3.2	-5.3	-2.0	-3.2	-2.7	-3.1	-6.3
Mdes008221	-	8.7	15.7	327.1	32.3	-	61.8	-	272.8	53.4
Mdes007867	-	-	7.5	249.6	24.4	-	22.3	4.4	476.5	10.7
Mdes006238	-2.3	-4.4	-2.2	-2.3	-	-1.6	-3.5	-	-2.8	-3.0
Mdes006146	7.6	45.9	7.9	47.0	24.0	3.7	16.5	2.5	15.7	6.2

A1, A3: Avirulent Biotype L larvae feeding on resistant Iris wheat at 1 and 3 DAH
V1, V3: Virulent Biotype L larvae feeding on susceptible Newton wheat at 1 and 3 DAH
Bd3: Biotype L larvae feeding on nonhost Bd plants at 3 DAH
