# Supplementary material for: Comparative Hessian Fly Larval Transcriptomics Provides Novel Insight into Host and Nonhost Resistance
Source: Int J Mol Sci. 2021 Oct 25;22(21):11498. doi: 10.3390/ijms222111498 (PMC8583952; doi:10.3390/ijms222111498)
Supplement: Supplementary file 1 [file ijms-22-11498-s001.zip › TableS8.pdf]

Table S8. Gene-specific primer sequences for validation of RNA-Seq expression data by qRT-PCR

Gene_id	Forward	Reverse
Mdes009774	CAATGGAGAGATTATGTCACCAGAA	TCGCAAACCCATCCATTTGT
Mdes018785	CGCCACCAAATGCTTACAGA	TTTCCTCGCTTTCGGTTCTC
Mdes009247	TGGGTTTGATGGTGTCTAAGGTTT	TGTTCGCCCAAAGAATAATTGA
Mdes018462	GGTTCAACAACACCAACCTATGC	ACACCATCCCAACGTTCATTC
Mdes009239	CGGCCCCATTTTTTGCA	AAAAACCGACCAAAACATCCTTT
Mdes010266	TCAACACATATCAATCGCCTGAT	ACATCGCATCCACGTTTGG
Mdes008221	CACGTGGTGTGCCCTTTCTT	TTGTGCATTCCGAAATTGATCT
Mdes007867	TTGAAATGACGGTCGACCTAAA	GAACCAGAAAACACAAAATTGCAT
Mdes006238	TCCAGCGTTCGCACAATTT	AATCATCTGGAGCCCCAAGA
Mdes006146	GTAGCGCATTCGGCCATAA	CGGTAAAATTGTTGCACGAAAA
